# Acclimation of subarctic vegetation to warming and increased cloudiness

**DOI:** 10.1002/pei3.10130

**Published:** 2023-11-28

**Authors:** Flobert A. Ndah, Marja Maljanen, Anne Kasurinen, Riikka Rinnan, Anders Michelsen, Titta Kotilainen, Minna Kivimäenpää

**Affiliations:** ^1^ Department of Environmental and Biological Sciences University of Eastern Finland Kuopio Finland; ^2^ Terrestrial Ecology Section, Department of Biology University of Copenhagen Copenhagen Ø Denmark; ^3^ Center for Volatile Interactions (VOLT), Department of Biology University of Copenhagen Copenhagen Ø Denmark; ^4^ Center for Permafrost (CENPERM), Department of Geosciences and Natural Resource Management University of Copenhagen Copenhagen K Denmark; ^5^ Natural Resources Institute Finland Turku Finland; ^6^ Natural Resources Institute Finland Suonenjoki Finland

**Keywords:** Arctic, climate change, cloud cover, temperature, tundra, vegetation change

## Abstract

Subarctic ecosystems are exposed to elevated temperatures and increased cloudiness in a changing climate with potentially important effects on vegetation structure, composition, and ecosystem functioning. We investigated the individual and combined effects of warming and increased cloudiness on vegetation greenness and cover in mesocosms from two tundra and one palsa mire ecosystems kept under strict environmental control in climate chambers. We also investigated leaf anatomical and biochemical traits of four dominant vascular plant species (*Empetrum hermaphroditum*, *Vaccinium myrtillus*, *Vaccinium vitis‐idaea*, and *Rubus chamaemorus*). Vegetation greenness increased in response to warming in all sites and in response to increased cloudiness in the tundra sites but without associated increases in vegetation cover or biomass, except that *E. hermaphroditum* biomass increased under warming. The combined warming and increased cloudiness treatment had an additive effect on vegetation greenness in all sites. It also increased the cover of graminoids and forbs in one of the tundra sites. Warming increased leaf dry mass per area of *V. myrtillus* and *R. chamaemorus*, and glandular trichome density of *V. myrtillus* and decreased spongy intercellular space of *E. hermaphroditum* and *V. vitis‐idaea*. Increased cloudiness decreased leaf dry mass per area of *V. myrtillus*, palisade thickness of *E. hermaphroditum*, and stomata density of *E. hermaphroditum* and *V. vitis‐idaea*, and increased leaf area and epidermis thickness of *V. myrtillus*, leaf shape index and nitrogen of *E. hermaphroditum*, and palisade intercellular space of *V. vitis‐idaea*. The combined treatment caused thinner leaves and decreased leaf carbon for *V. myrtillus*, and increased leaf chlorophyll of *E. hermaphroditum*. We show that under future warmer increased cloudiness conditions in the Subarctic (as simulated in our experiment), vegetation composition and distribution will change, mostly dominated by graminoids and forbs. These changes will depend on the responses of leaf anatomical and biochemical traits and will likely impact carbon gain and primary productivity and abiotic and biotic stress tolerance.

## Graphical abstract

Climate warming will likely occur in combination with increased cloudiness in the high latitude regions and may result in a host of ecosystem and plant species changes, including changes in vegetation structure and composition. These changes may have consequences for carbon gain and primary productivity, and abiotic and biotic stress tolerance.

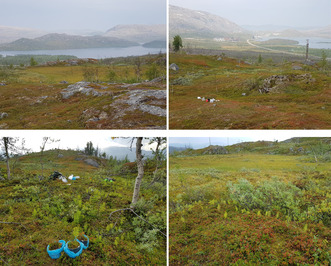

## 1 INTRODUCTION

The high latitudes of the Northern hemisphere are warming at a faster rate compared to the global average rate, a phenomenon known as Arctic amplification, with impacts occurring at a magnitude and pace unprecedented in recent history (IPCC, 2022). Over the last three decades, the Arctic has experienced a temperature increase of about 1°C and the projected increase in mean annual temperature may be about +4°C by mid‐century (Overland et al., 2019). Projected summer air temperature increase is expected to accelerate the melting of the ice cover in these high latitude regions (Overland et al., 2019). This may lead to increased cloud cover (Liu et al., 2012) due to enhanced latent heat fluxes, moisture release and atmospheric accumulation of aerosol particles from the ice‐free surfaces, such as glacial retreat (Kecorius et al., 2019; Sedlar et al., 2011). Furthermore, models project an increase in the poleward movement of clouds and the maximum height of cloud formations during recent warming (Norris et al., 2016). While generally expected to have a net cooling effect (Kulmala et al., 2013; Scott et al., 2014; Shindell et al., 2009) associated with a decrease in incoming solar radiation (Kejna et al., 2021), clouds in these high latitude regions have the potential to warm the Earth's surface (Intrieri et al., 2002; Kay & L'Ecuyer, 2013; Norris et al., 2016; Zygmuntowska et al., 2012) through their positive cloud radiative effect (Sedlar et al., 2011) thereby enhancing Arctic amplification. For example, thinner clouds with smaller optical depth and lower albedo effect may enhance Arctic amplification and cloud formation by transmitting much of shortwave radiation and emitting absorbed longwave radiation to the Earth's surface (Bennartz et al., 2013; Cotton et al., 2011). As the optical depth increases (thicker clouds), the albedo effect increases resulting in enhanced decrease in incoming solar radiation and net cooling of the Earth's surface, but still with the potential of enhancing Arctic amplification via enhanced longwave radiative flux at the surface by thicker lower clouds (Cotton et al., 2011; Huang et al., 2021).

The projected future changes, warming and increased cloud cover, can affect a vast number of ecosystem responses. Warming has been linked to widespread changes in tundra vegetation, for example, increase in vegetation greening, aboveground plant biomass and productivity (May et al., 2017, 2020), increase in height and abundance of shrubs and graminoids, and decrease in the cover of lichens, moss, and bare ground surfaces (Cornelissen et al., 2001; Elmendorf, Henry, Hollister, Björk, Bjorkman, et al., 2012; Elmendorf, Henry, Hollister, Björk, Boulanger‐Lapointe, et al., 2012; Hollister et al., 2015; Myers‐Smith et al., 2011; Pearson et al., 2013; Walker et al., 2006). Lower light intensities via increased cloudiness in a changing climate or via shading by expanding canopy‐forming taller shrubs (Myers‐Smith et al., 2011, 2020) have mostly been reported to decrease vegetation greenness (Dahl et al., 2017; May et al., 2021) and aboveground plant biomass and productivity (Chapin et al., 1995; Chapin & Shaver, 1996; Dahl et al., 2017; Graglia et al., 1997; Körner, 1982; Zhang & Welker, 1996).

Leaf anatomical and biochemical acclimation to warming and increased cloudiness (Baruah et al., 2017; Bjorkman et al., 2018; Hansen et al., 2006; Schollert et al., 2015, 2017; Sundqvist et al., 2011) can contribute to the abovementioned vegetation responses and ecosystem functioning (Lavorel & Garnier, 2002). For example, alterations in leaf anatomical traits, such as thickness of leaf tissues, density of stomata and trichomes, affect plant's photosynthetic gas exchange, water relations, and abiotic and biotic stress tolerance (Larcher, 2003; Luomala et al., 2005), and thus, growth and survival. Leaf biochemical traits such as chlorophyll and nitrogen (N) content affect primary productivity, litter decomposability, soil carbon storage, herbivory, and nutrient cycling (Cornelissen et al., 2007; Cornwell et al., 2008; Hansen et al., 2006; Hudson et al., 2011).

As climate change in the Arctic and Subarctic regions is expected to have substantial effects on plant distribution, abundance, species richness and biodiversity (Callaghan et al., 2005; Moffat et al., 2016; Myers‐Smith et al., 2011), studies that examine plant community composition and species level trait responses to environmental factors are needed to refine our understanding of consequences of these responses on ecosystems (Hudson et al., 2011; Suding et al., 2008). The long and short‐term effects of warming and shading (simulating increased cloudiness) on (sub)arctic vegetation cover and greenness, and leaf anatomy and biochemistry have been reported (Dahl et al., 2017; Hollister et al., 2015; Hudson et al., 2011; May et al., 2020; Ndah et al., 2022; Schollert et al., 2015, 2017; Tolvanen & Henry, 2001; Walker et al., 2006). However, the previous studies have mostly applied less pronounced warming treatments than currently projected for Subarctic ecosystems and simulations of cloud cover have been based on hessian tents that reduces a certain percentage of ambient radiation/photosynthetic active radiation (PAR). Also, information is lacking on the combined effects of warming and increased cloudiness.

Our aim was to provide new information about the combined effects of warming and increased cloudiness on vegetation compositional distribution and greenness, and leaf anatomical and biochemical traits of four dominant vascular plant species in Subarctic ecosystems. We hypothesized that warming would lead to an increase in vegetation greenness and an associated increase in the cover and biomass of shrubs and graminoids as opposed to lichens and mosses (May et al., 2017, 2020; Walker et al., 2006). Furthermore, warming was expected to cause thicker leaves and increased stomata and trichome densities (Schollert et al., 2015, 2017), decreased leaf N (Tolvanen & Henry, 2001), and increased leaf carbon (C) and chlorophyll concentrations (Michelsen et al., 1996). Reduced light availability simulating increased cloudiness was expected to have an opposite effect resulting in decreased vegetation greenness (Dahl et al., 2017; May et al., 2021), hence decreased cover and biomass of shrubs and graminoids (Dahl et al., 2017; Graglia et al., 1997; Zhang & Welker, 1996) as opposed to lichens and mosses. Reduced light availability was also expected to cause thinner leaves and reduced stomata and trichome densities (Larcher, 2003; Marques et al., 1999), decreased leaf C, and increased leaf N and chlorophyll concentrations (Hansen et al., 2006; Iason & Hester, 1993; Michelsen et al., 1996). Since warming and increased cloudiness are expected to have opposite effects, we assumed that they would largely counterbalance the effects of each other in the combined treatment.

## 2 MATERIALS AND METHODS

### 2.1 Plant material and sampling of mesocosms

In August 2019, 60 soil cores/mesocosms, that is, blocks of soil and intact vegetation on top, were collected from three subarctic sites (20 mesocosms per site). The collection sites included a heath tundra close to Vassijaure weather station (68°25′45″ N, 18°15′37″ E, 550 m above sea level) in Northern Sweden and a palsa mire in Kilpisjärvi (68°53′4.5″ N, 21°3′10.7″ E), Northern Finland. Mesocosms were collected from two locations in the tundra site that appeared different in soil fertility based on vegetation; the more fertile site was close to the upper border of mountain birch tree line hereafter referred to as the tundra1 (T1) site while the less fertile site was uphill from the T1 site, about 100 m apart, hereafter referred to as the tundra2 (T2) site. The palsa mire site is hereafter referred to as the palsa (P) site. The tundra sites in Vassijaure were characterized by podsol soil formation while the palsa site in Kilpisjärvi was a peatland, with several meters of peat.

Mesocosms were collected by cutting and digging out a piece of soil containing plants into 18 × 15 cm (diameter × height) polyvinyl chloride (PVC) rings. The PVC rings were hammered into the soil and the rings with soil were removed carefully with a spade and placed over an aluminum plate. The vegetation cover at the collection sites and in the mesocosms consisted of a mixture of various evergreen and deciduous dwarf shrubs (e.g., *Empetrum hermaphroditum*, *Vaccinium vitis‐idaea*, *Vaccinium myrtillus*), graminoids (e.g., *Deschampsia flexuosa*, *Carex vaginata*), forbs (e.g., *Cornus suecica*, *Rubus chamaemorus*), mosses (e.g., *Pleurozium schreberi*), and lichens (e.g., *Nephroma arcticum*, *Cladonia arbuscula*). The mesocosms were heterogeneous; some with very dense and others with less vegetation cover. Vegetation composition of mesocosms from the tundra sites (T1 and T2) was dominated by deciduous shrubs, graminoids, and forbs, with mosses and lichens covering the ground layer. Based on vegetation, T1 appeared more fertile than T2 with more graminoids and forbs but less lichen cover (Table S1; Figure S1). Mesocosms from the P site were dominated by evergreen shrubs with the ground layer mostly composed of litter and patches of bare peat (Table S1; Figure S1). After collection, mesocosms were weighed and transported to the laboratory and regularly sprayed with distilled water to avoid desiccation. Plant dormancy occurring in (sub)arctic winter under snow was achieved by moving the mesocosms to a well‐ventilated dark room, where they were maintained at +4°C and regularly sprayed, prior to exposure to experimental climate treatments in the beginning of June 2020.

### 2.2 Experimental setup and climate simulation

The experimental design had four treatments: control, warming, increased cloudiness, and warming + increased cloudiness. Twenty mesocosms from each site (T1, T2, P) were randomly divided into four groups and placed in four respective climate chambers (Fitotron, WEISS Technik UK LTD) for the simulation of the actual and future climates. Each climate treatment had five replicate mesocosms from each ecosystem site giving a total of 15 mesocosms per climate treatment. The chambers were equipped with 2 × Valoya G2 + 4 × Valoya NS1 LED luminaires (Valoya B100, Finland) at a height of 30 cm from the top of plant canopy. A mesh fabric (SEFAR NITEX® 03‐50/37, Switzerland) was placed between the lamps and plant canopy top (about 5 cm below lamps and 25 cm above plant canopy top) to create diffuse light environment in the chambers since the northern high latitudes experience predominantly diffused rather than direct light due to the presence of frequent clouds coupled with atmospheric particles or pollutants. See Table S2 for PAR and spectral composition of simulated irradiances for Vassijaure and measured irradiances from Valoya luminaires in the climate chambers.

Climate data were collected from the Katterjåkk weather station (68°24′45″ N, 18°08′13″ E), which is closest to Vassijaure with similar climatic conditions. We simulated the summer climate in the control chamber/treatment by using the 2010–2019 weekly average climate data of June, July, and August in Katterjåkk and increased the air temperatures by 4°C in the warming chambers (Figures S2–S13). Temperature increase of 4°C was selected as it presents an average increase of several climate change models based on the Representative Concentration Pathway (RCP) 4.5 scenario for the year 2100 in the Scandinavian Subarctic. Irradiance conditions in the climate chambers were based on simulations of PAR and how it changes on a weekly basis according to the time of day, that is, solar elevation angle and cloud thickness during the months of June, July, and August in Katterjåkk. Simulations were done with the *uvspec* model from libRadtran, version 2.0.1, radiative transfer package (Emde et al., 2016), as described in detail in Kotilainen et al. (2020). Control climate treatment was based on simulations with cloud optical depth of ten representing thin‐cloud cover conditions (Table S2; Figures S2–S13). The increased cloudiness treatment had 50% decrease in PAR compared to the control representing thick‐cloud cover conditions (cloud optical depth of thirty) (Figures S2–S13).

Mesocosms were exposed to the summer climate treatments in the chambers during two consecutive years (2020 and 2021) and for 3 months (June–August) each year, averaging the length of the main growing season in the Subarctic. To minimize chamber effects, mesocosms were rotated within chambers on a weekly interval and mesocosms and treatments rotation between chambers was done on bi‐weekly intervals. The initial mesocosm weight was maintained by watering with distilled water. At the end of the first growing season, mesocosms were stored at +4°C in a well‐ventilated dark room, and regularly sprayed, to achieve plant dormancy occurring in Subarctic winter under snow prior to the start of the second growing season in 2021.

### 2.3 Vegetation analyses

#### 2.3.1 Vegetation cover

At the start (pre‐exposure to experimental treatments) and end (post‐exposure to experimental treatments) of the first growing season, mesocosm vegetation cover (%) was determined using visual estimation (Mäki et al., 2017). Briefly this was done by assessing the percentage of the ground or soil surface in each mesocosm that was covered by the plant species. During the second growing season, vegetation cover (%) was estimated four times using the point intercept method (Jonasson, 1988). Briefly, a pin was passed through a grid with 16 intersects placed above each mesocosm and the species was recorded each time it was touched by the pin. All identified plant species were assigned to the functional groups: evergreen shrubs, deciduous shrubs, graminoids, forbs, mosses, and lichens. We also estimated the percentage cover of litter + bare soil at the end of the first growing season and of litter, standing dead vegetation (dead vegetation with root connections), and bare soil during the second growing season.

#### 2.3.2 Vegetation greenness

We adapted the method of Liang et al. (2017) to obtain an estimate of vegetation greenness in each mesocosm during the second growing season. The method provides a convenient and non‐destructive way of estimating changes in vegetation pigment concentration over time using calibrated RGB images. True‐color images of each mesocosm were acquired bi‐weekly, eight times in total across the second growing season (i.e., 4 June, 16 June, 30 June, 14 July, 27 July, 12 August, 21 August, and 31 August) using a consumer‐grade digital camera (Olympus Stylus TG‐4 Tough, Tokyo, Japan). Settings for the digital camera were the following: picture mode = natural, no flash, exposure compensation (exposure comp) = ±0.0, white balance (WB) = automatic (AUTO), autofocus (AF) mode = single‐point focus, aperture = f/8, Shutter speed = 1/100 s, ISO = 400, file format = RAW. Images were taken at about 29 cm to the upper edge of mesocosm cores and under uniform light conditions in a light tent (Godox LSD40, China). The light tent was equipped with LED lamps, which were set to full power, giving PAR of 150 μmol m^−2^ s^−1^. To ensure all acquired images were comparable, images were converted from RAW (ORF file) format to DNG format and calibrated using DNG profile of an X‐rite Color‐Checker Classic Mini Target (X‐rite Pantone, USA) generated through Adobe Photoshop/Lightroom software. Images were then exported in JPEG format and the background, other than plants and patches of bare soil and litter in some mesocosms, was eliminated using crop tool in ImageJ (1.47 v, Wayne Rasband, National Institutes of Health, Bethesda, Maryland, USA). RGB values of each pixel in cropped images were extracted using ImageJ. Based on the RGB values of our digital images, the RGB indices were calculated according to Beamish et al. (2018). These indices, greenness index (nG) (nG = G/G + R + B) and greenness excess index (GEI) (GEI = 2G‐RB, Richardson et al., 2007) were used to monitor seasonal vegetation changes associated with pigment‐driven color changes, mostly related to the amount of green or chlorophyll pigments. Indices nG and GEI can be used to monitor changes in vegetation phenology and productivity (Beamish et al., 2016; Ide & Oguma, 2013; Richardson et al., 2007), but GEI provides a more sensitive indicator of changes in plant pigments (Ide & Oguma, 2013), and is desirable in tundra ecosystems where changes can be subtle (Beamish et al., 2016).

#### 2.3.3 Leaf anatomy, chlorophyll, C, and N content, and total biomass

Sampling for leaf anatomy, leaf chlorophyll, C, and N content, and total biomass was done during the second growing season to evaluate the cumulative effects of the treatments. Individual shoots of four dominant vascular plant species in most of the experimental mesocosms from at least two sites were chosen for the analyses of leaf anatomy and chlorophyll content, involving two evergreen shrubs *E. hermaphroditum* and *V. vitis‐idaea*, the deciduous shrub *V. myrtillus* and the forb *R. chamaemorus*. *E. hermaphroditum* was sampled from mesocosms of T1, T2, and P sites (*n* = 6–11 per treatment), *V. vitis‐idaea* from T2 and P (*n* = 6–9), *V. myrtillus* from T1 and T2 (*n* = 6–11), and *R. chamaemorus* from T1 and P (*n* = 4–6).

##### Leaf anatomy

Sample collection for light microscopy (LM) and scanning electron microscopy (SEM) was done during the second growing season, on 28 June, for *V. myrtillus* and *R. chamaemorus*. *E. hermaphroditum* and *V. vitis‐idaea* samples were collected later during the growing season, on 11 August, when their new leaves and shoots were fully developed. For *R. chamaemorus*, a fully developed leaf from the main shoot of one plant per mesocosm, and for *V. myrtillus* and *V. vitis‐idaea* two second or third fully developed leaves from top of the branch from two different branches/ramets per plant per mesocosm were carefully detached with forceps. For *E. hermaphroditum* two leaves from middle of the current year shoot (ca. 1 cm from the tip) from two different shoots were detached.

After collection, leaf samples from all species, except for *E. hermaphroditum*, were cut along the midrib of which one half of each leaf was used for LM and the other half for SEM. Samples for LM were cut in smaller sections the same day. For *R. chamaemorus, V. myrtillus*, and *V. vitis‐idaea*, two ca. 1 × 1.5 mm sections were cut next to the midrib while for *E. hermaphroditum*, the 1.5 mm long segments were cut from the middle part of the leaf. Leaf sections were put into cold (+4°C) prefixative containing 2.5% glutaraldehyde (Electron Microscopy Sciences, Hatfield, PA, USA) in 1 M phosphate buffer (pH 7.0) overnight.

Samples were processed the next day with Lynx Microscopy Tissue Processor (Reichert‐Jung Optische Verke AG, Wien, Austria) as follows: rinsed with the 0.1 M phosphate buffer (3 × 10 min, +4°C), post‐fixed in 1% buffered (same as in prefixative) osmium tetroxide (Electron Microscopy Sciences, Hatfield, PA, USA) for 3 h for *R. chamaemorus*, *V. myrtillus*; 5 h for *V. vitis‐idaea*, *E. hermaphroditum* (+4°C), rinsed with the buffer (3 × 10 min, +4°C), dehydrated in increasing ethanol series (50%, 70%, 94%, 100%, 2 × 10 min each, +4°C), treated with propylene oxide (Sigma‐Aldrich, Steinheim, Germany) for 3 × 10 min (+20°C); mixture of propylene oxide:epon (Ladd LX112, Burlington, Vermont, USA) (3:1) for 1 h (+20°C), propylene oxide:epon 1:1 for 2 h (+20°C), propylene oxide:epon 1:3 overnight (+20°C) and pure epon overnight (+20°C). The samples were embedded in Ladd's epon in flat embedding molds made of silicon (Electron Microscopy Sciences, Hatfield, PA, USA). Semithin (1 μm) sections for LM were cut from the embedded samples using an ultramicrotome (Reichert‐Jung Ultracut E, Wien, Austria, Diatomen histo‐knife [Hi 4967]) and stained with toluidine blue as described by Kivimäenpää et al. (2010).

**FIGURE 1:**
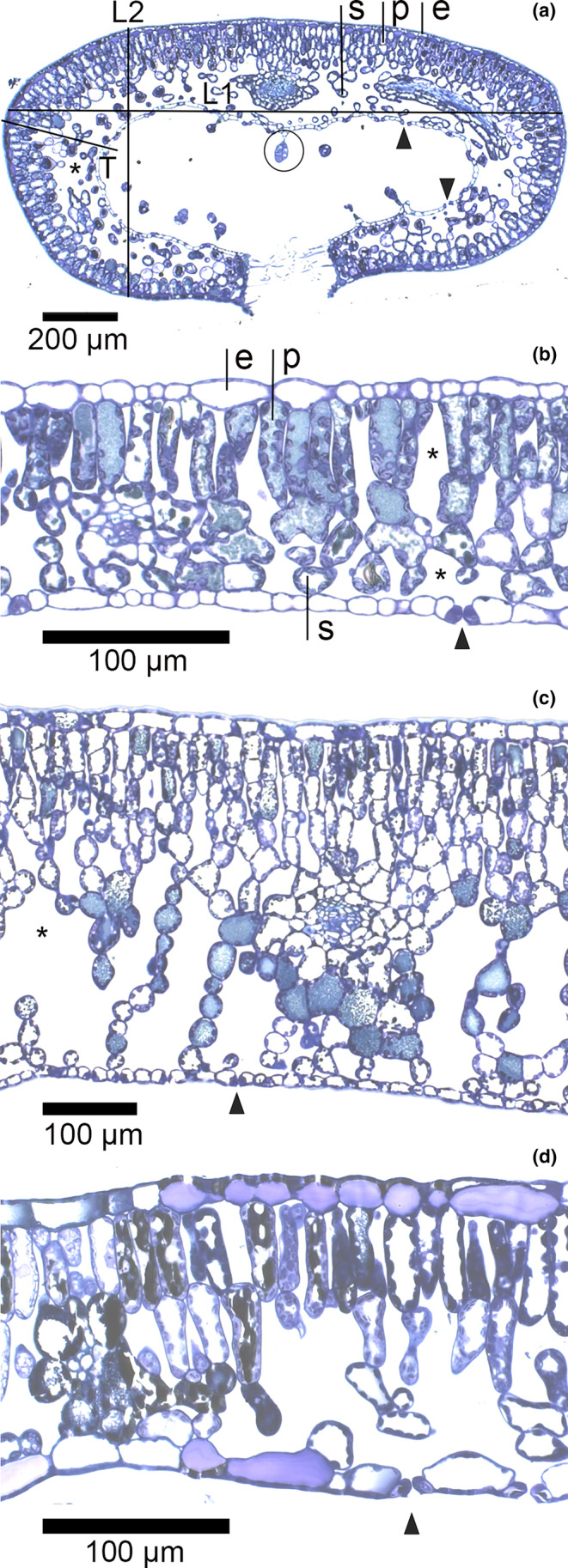
Light microscopy images of cross‐section for (a) *Empetrum hermaphroditum*, (b) *V. myrtillus*, (c) *V. vitis‐idaea* and (d) *R. chamaemorus*. In (a) L1 and L2 show leaf dimensions to calculate shape index (L1:L2), T indicates location for leaf and tissue thicknesses measurement. Glandular trichome is encircled. Note that stalks are not visible for all glandular trichomes. p = palisade tissue, s = spongy tissue, e = outer (a) or upper epidermis (b). Arrowheads indicate stomata in inner (a) or lower epidermis (b–d). Asterisks indicate intercellular space.

LM sections were studied with a light microscope (Zeiss Primo Star light microscope, Jena, Germany) and photographed (Zeiss Axiocam ERc 5s camera, Jena, Germany). The objective magnification varied for the species. The following leaf anatomical parameters were determined for all four studied species: leaf thickness, epidermis thickness, palisade and spongy parenchyma thicknesses, proportion of intercellular space in palisade and spongy tissues, and shape change index for *E. hermaphroditum*, as described in Figure 1a. Leaf thickness of *E. hermaphroditum* was measured as described in Figure 1a. Leaf margins of *E. hermaphroditum* are curved forming a cavity in the middle of leaves, where the stomata are found (Figure 1a). Thus, the terms inner and outer surface is used for them, corresponding to lower and upper surface, respectively, in the other studied species. The other studied species are broadleaved with less complex leaves having adaxial (upper) palisade parenchyma layer and abaxial (lower) spongy parenchyma layer (Figure 1b–d). Glandular trichomes (GTs) and stomata density (#/mm^−1^) were measured per inner leaf surface length for *E. hermaphroditum*.

The other half of the leaf samples for SEM were air‐dried at room temperature, cut into pieces that fitted on double‐sided copper tape on aluminum stubs and sputtered with ca. 50 nm layer of gold (Automatic Sputter Coater B7341, Agar Scientific Ltd., Stansted, UK). The samples were examined using SEM (Philips XL30 ESEM‐TMP, FEI Company, Eindhoven, Netherlands) and digitally photographed using 80× magnification for *R. chamaemorus* and *V. vitis‐idaea* and 60× magnification for *V. myrtillus* to analyze trichome densities and 500× magnification for *R. chamaemorus*, *V. vitis‐idaea* and *V. myrtillus* to analyze stomata densities. All analyses of digital images were done using ImageJ (1.47 v, Wayne Rasband, National Institutes of Health, Bethesda, Maryland, USA).

Leaf dry mass per area (LMA) was determined for the broadleaved species *V. myrtillus*, *V. vitis‐idaea*, and *R. chamaemorus* at the end of the second growing season. From each sample mesocosm containing these plant species, 5 leaves for *V. myrtillus* and 1–3 leaves for *V. vitis‐idaea* and *R. chamaemorus* were collected from different ramets/plants. Collected leaves were scanned in a flatbed scanner (HP Scanjet 3670, China) and the leaf areas (LA) were analyzed using ImageJ. Oven‐dried leaves (60°C for 2–3 days) were weighed to obtain the dry mass and to calculate LMA by dividing with LA.

##### Leaf chlorophyll, C, and N content

Leaf chlorophyll content, as a proxy for photosynthesis, was measured from 2 to 3 undamaged ramets/shoots/leaves of the main vascular plant species *E. hermaphroditum*, *V. myrtillus*, *V. vitis‐idaea*, and *R. chamaemorus* during the second growing season (5 and 6 July) using a portable chlorophyll content meter (CCM‐300, Opti‐Sciences, Inc. 8 Winn Ave. Hudson, NH 03051, USA). For *E. hermaphroditum*, measurement was taken by placing the sensor at the side against the tip of the shoot. For *V. myrtillus*, measurement was taken from the lower surface of fully developed third leaf from the top of the ramet and for *V. vitis‐idaea* from the upper surface of fully developed leaf from the top of the ramet. For *R. chamaemorus*, measurements were also taken from the upper surface of the leaf. Different measurement methods such as upper and lower side measurements were employed to ensure minimal damage to the leaves.

C and N content from leaves of *E. hermaphroditum* and *V. myrtillus*, which were more abundant and yielded sufficient sample quantity for the analysis compared to the other main vascular plant species *V. vitis‐idaea* and *R. chamaemorus*, were also measured at the end of the second growing season. Leaf samples were oven‐dried at 60°C for 2–3 days, homogenized, and analyzed using an Isoprime isotope ratio mass spectrometer (Isoprime Ltd, Cheadle Hulme, Stockport, UK) coupled to a CN elemental analyzer (Eurovector, Milan, Italy) with continuous flow.

##### Total biomass

Mesocosms were harvested for above and below ground biomass at the end of the 2‐year experiments in September 2021. The aboveground parts containing current (2021) and previous (2020) year shoots/leaves and stems of the main vascular plant species *E. hermaphroditum*, *V. myrtillus*, *V. vitis‐idaea* and *R. chamaemorus* per mesocosm were harvested and placed in separate paper bags. Aboveground parts of plant species grouped as other evergreen shrubs (e.g., *Phyllodoce caerulea*, *Andromeda polifolia*, *Kalmia procumbens*), other deciduous species (e.g., *Vaccinium uliginosum*, *Betula nana*, *Arctostaphylos alpina*, *C. suecica*, *Pedicularis lapponica*, *Cerastium alpinum*), graminoids (e.g., *D. flexuosa*, *C. vaginata*), mosses (e.g., *P. schreberi*, *Polytrichum commune*, *Hylocomium splendens*, *Dicranum* spp., *Sphagnum* sp.), lichens (e.g., *N. arcticum*, *C. arbuscula*, *Cladonia* spp., *Thamnolia vermicularis*, *Stereocaulon paschale*), and non‐photosynthetic plant materials (e.g., litter, roots, and standing dead vegetation, that is, dead vegetation with root connection) were also harvested. Plant parts were oven‐dried at 60°C for 2–3 days after which their dry biomass were measured.

### 2.4 Analyses of soil physiochemical properties

The organic layer of the soil was carefully separated from the mineral layer (present only in T1 and T2) and the depth of the organic layer was measured. The organic soil was homogenized, sieved with a 5 mm mesh sieve, and stored at +4°C until analyses. The organic matter content was determined by accounting for the residue on ignition, loss on ignition and dry weight of the soil. Bulk density was determined by dividing the weight of soil by the volume of soil. Mineral N was extracted from the soil samples with milliQ‐H_2_O for nitrite (${\mathrm{NO}}_{2}^{-}$) and nitrate (${\mathrm{NO}}_{3}^{-}$) analyses and with 1 M KCl for ammonium (${\mathrm{NH}}_{4}^{+}$) analysis. All extracts were stored at +4°C prior to analyses. Concentrations of ${\mathrm{NO}}_{2}^{-}$ and ${\mathrm{NO}}_{3}^{-}$ from soil extracts were analyzed with an ion chromatograph (IC, Thermo Scientific, Dionex ICS‐2100) coupled to an autosampler (Thermo Scientific, Dionex AS‐DV). The ${\mathrm{NH}}_{4}^{+}$ concentrations from soil extracts were analyzed spectrophotometrically at 650 nm with the colorimetric method as described in Fawcett and Scott (1960). Soil pH and electrical conductivity (EC) were measured from the soil slurry prepared for ${\mathrm{NO}}_{2}^{-}$ and ${\mathrm{NO}}_{3}^{-}$ extraction with pH meter (WTW, pH 340) and EC meter (WTW pH/cond 340i), respectively. Soil C and N content were measured using elemental C and N analyzer (varioMax CN elemental analyzer, Elementar Analysensystem GmbH, Germany) while total organic C content was analyzed with TOC analyzer (TOC Analyzer, SHIMADZU Corporation) from the milliQ‐H_2_O extracts used for anion (${\mathrm{NO}}_{2}^{-}$ and ${\mathrm{NO}}_{3}^{-}$) analysis. Gravimetric soil moisture content (GWC) was determined by drying the samples for 48 h at 65°C. Water holding capacity (WHC) was also determined.

### 2.5 Statistical analyses

Univariate analysis of variance (ANOVA) (IBM SPSS Statistics 27.0.0, SPSS Inc. IBM Company©, Armonk, NY, USA) was used to test the main and interaction effects of warming and increased cloudiness on leaf anatomy, leaf chlorophyll, C, and N content, and total biomass parameters. Warming and increased cloudiness were included as fixed factors in the model. The effects of warming, increased cloudiness, site, time and their interactions on nG and GEI were tested using linear mixed models (LMM) ANOVA. The model included warming, increased cloudiness, site, and time as fixed factors whereas random factor included mesocosms as subjects. In addition, repeated measures type of setting was accounted with time. The final model was obtained by excluding nonsignificant (cutoff level 0.2) effects, one by one, starting from the highest‐level interactions and highest probability values (Underwood, 1996). Selection of covariance structure was based on the smallest Akaike's information criteria (AIC) and was set to either Diagonal (DIAG), Heterogenous compound symmetry (CSH) or Heterogenous autoregressive [ARH (1)]. In all analyses, interactions *p* < .1 were further studied by calculating *p*‐values for simple main effects (SME, i.e., post hoc test for interactions) with Bonferroni corrections. The normality of the data and the model residuals for LMM ANOVA were checked by the Shapiro–Wilk's normality test and by generating normality plots (histograms). In case data were not normally distributed, or showed inhomogeneous variances, logarithmic or square root transformations were made. Variables that did not fulfill the prerequisites of ANOVA after transformations were tested using the Kruskal–Wallis test. In all analyses, statistical significance was considered at the *p* < .05 level.

Principal component analysis (PCA) in SPSS was used to evaluate differences in vegetation composition data (percentage cover of plant functional groups) prior to exposure to experimental treatments in the first growing season. PCA was also used to summarize the vegetation composition data measured at the end of the first growing season (percentage cover of plant functional groups) and at different intervals during the second growing season (average percentage cover of plant functional groups) into principal components that were then tested for main and interaction effects of warming, increased cloudiness, and site. In all PCA analyses, the scores of the first two components generated were tested using similar univariate ANOVA models as described earlier. Correlations between leaf or soil C and N content and leaf anatomy, and soil C and N content and total biomass were analyzed using the Spearman's Rank‐Order Correlation test.

## 3 RESULTS

### 3.1 Soil physiochemical properties

Soils from the three study sites were acidic (pH 3.6–4.0). The T2 site had a slightly higher bulk density (BD) and lower WHC than the T1 and P sites (Table 1). T1 and P sites had highest and lowest TOC concentrations, respectively, and showed the opposite pattern for ${\mathrm{NH}}_{4}^{+}$ (Table 1).

**TABLE 1:** Mean (±SE, *n* = 20 per site) physiochemical properties of organic soils from mesocosms in tundra1 (T1), tundra2 (T2), and palsa (P) sites.

	T1	T2	P
Depth of organic layer (cm)	6.7 ± 0.7	4.1 ± 0.3	11.2 ± 0.1
Organic matter content (OM) (%)	87.0 ± 2.3	86.0 ± 2.1	96.7 ± 0.2
pH	4.0 ± 0.1	3.9 ± 0.1	3.6 ± 0.03
Electrical conductivity (EC) (μS cm^−1^)	71.0 ± 2.4	71.8 ± 5.5	75.6 ± 2.4
Bulk density (BD) (g cm^−3^)	0.5 ± 0.02	0.6 ± 0.03	0.4 ± 0.02
Water holding capacity (WHC) (%)	709.7 ± 18.7	601.0 ± 16.4	821.5 ± 67.7
Gravimetric water content (GWC) (%)	14.8 ± 20.0	12.7 ± 20.0	24.0 ± 20.0
Nitrite (${\mathrm{NO}}_{2}^{-}$) (μg g^−1^)	0.6 ± 0.1	0.4 ± 0.1	0.6 ± 0.1
Nitrate (${\mathrm{NO}}_{3}^{-}$) (μg g^−1^)	0.3 ± 0.1	0.7 ± 0.1	0.6 ± 0.2
Ammonium (${\mathrm{NH}}_{4}^{+}$) (μg g^−1^)	6.6 ± 1.9	12.1 ± 3.5	21.2 ± 7.9
Total organic carbon (TOC) (μg g^−1^)	450.0 ± 20.6	343.0 ± 22.6	261.8 ± 25.5
Carbon (C) (%)	42.0 ± 0.6	42.8 ± 0.5	45.7 ± 0.4
Nitrogen (N) (%)	1.5 ± 0.05	1.4 ± 0.1	1.3 ± 0.1

*Note*: Average organic layer depth of mesocosms from T1, T2, and P sites are shown. Organic layer depth for T1 and T2 sites are representative of the field site conditions unlike the P site where the organic layer at field site was several meters thick.

### 3.2 Vegetation cover, biomass, and greenness

#### 3.2.1 Vegetation cover and biomass

Across all sites and treatments in both years, mesocosm vegetation composition was dominated by evergreen and deciduous shrubs followed by graminoids and forbs with mosses and lichens covering the ground layer (Tables S3 and S4). The most dominant evergreen shrubs were *E. hermaphroditum* and *V. vitis‐idaea* while the deciduous shrubs were dominated by *V. myrtillus*. The graminoids were dominated by *D. flexuosa* while the forbs present were mainly *R. chamaemorus* and *C. suecica*. The most dominant mosses were *P. schreberi* followed by *Dicranum* spp. while the dominant lichens were *Cladonia* spp. and *N. arcticum*.

At the end of the first growing season, PCA on the vegetation cover data revealed a significant effect of increased cloudiness along PC1 (Figure S14). Increased cloudiness increased the cover of mosses (*P. schreberi* and *Dicranum* spp.) compared to the control treatment with an opposite effect for evergreen shrubs (*E. hermaphroditum* and *P. caerulea*) (Figure S14; Table S3). A warming × site (W × S) and increased cloudiness × site (‐PAR × S) interaction on the percentage cover of deciduous shrubs, lichens, and litter + bare soil along PC2 was detected (Figure S14; Table S5). Warming and increased cloudiness, acting independently, decreased the cover of deciduous shrubs (*V. myrtillus*) and lichens (*N. arcticum*, *C. arbuscula*, *Cladonia* spp., *S. paschale* and *T. vermicularis*) for the T2 site (Figure S14; Tables S5 and S6).

**FIGURE 2:**
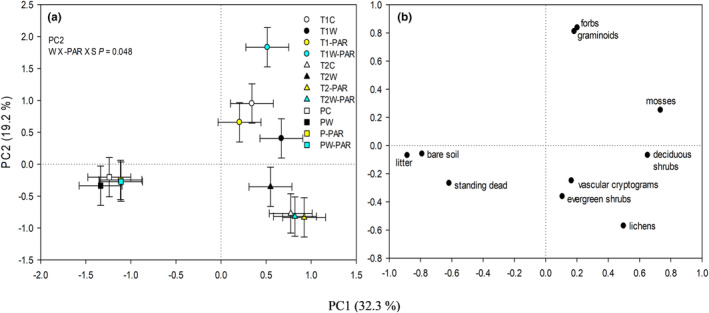
(a) The principal component (PC) scores (means ± S.Es) and (b) their respective loading variables for percentage cover of plant species functional groups, litter, standing dead and bare soil in the second growing season. The variation explained by each PC is shown in parentheses. T1C = tundra1 site in control (ambient warming and PAR/thin cloud), T1W = tundra1 site in warming, T1‐PAR = tundra1 site in increased cloudiness, T1W‐PAR = tundra1 site in warming + increased cloudiness, T2C = tundra2 site in control (ambient warming and PAR/thin cloud), T2W = tundra2 site in warming, T2‐PAR = tundra2 site in increased cloudiness, T2W‐PAR = tundra2 site in warming + increased cloudiness, PC = palsa site in control (ambient warming and PAR/thin cloud), PW = palsa site in warming, P‐PAR = palsa site in increased cloudiness, PW‐PAR = palsa site in warming + increased cloudiness treatments. The univariate ANOVA *p*‐value for statistically significant (*p* ≤ .05) interaction effect is shown in the figure (*n* = 5 per site per treatment).

In the second growing season, a warming × increased cloudiness × site (W × ‐PAR × S) interaction on the percentage cover of plant species functional groups along PC2 was detected (Figure 2). Mesocosms from the T1 site in the combined warming + increased cloudiness treatments showed a higher cover of graminoids (*D. flexuosa*), and forbs (*C. suecica*) compared to those in the only warming or increased cloudiness treatments (Figure 2; Tables S5 and S7).

**FIGURE 3:**
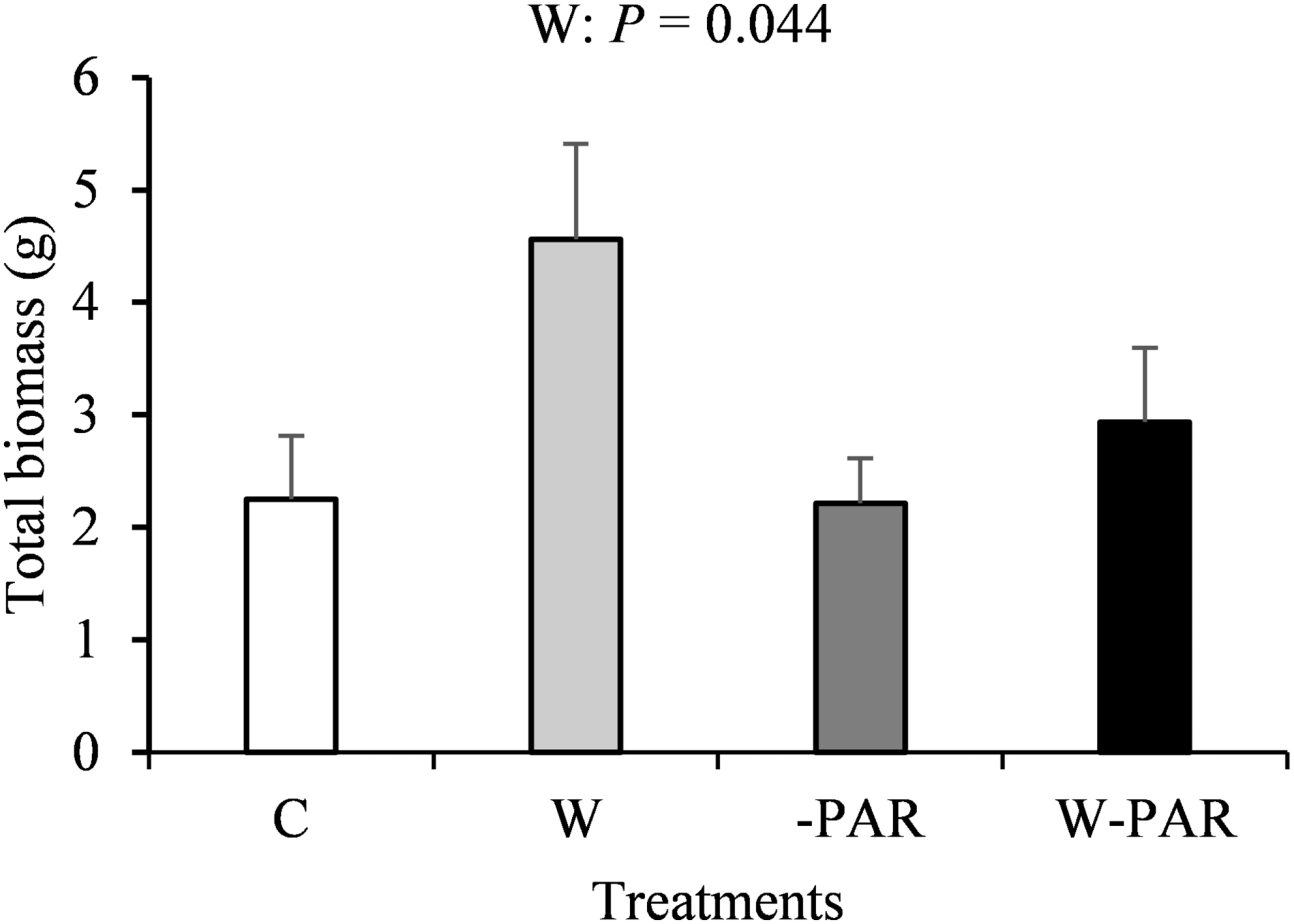
Total biomass (mean ± SE, *n* = 11–13 per treatment) of *Empetrum hermaphroditum* in control (C), warming (W), increased cloudiness (‐PAR), and warming + increased cloudiness (W‐PAR) treatments. *p*‐value <.05 for main effect of W from univariate ANOVA is shown.

Warming significantly increased total biomass of *E. hermaphroditum* (Figure 3) but not of the main vascular plant species *V. myrtillus*, *V. vitis‐idaea* and *R. chamaemorus* (Figure S15a–c). There were no treatment effects on total biomass of other plant species or functional groups, litter, standing dead, roots, and other non‐photosynthetic plant parts (Figures S16a–f and S17a,b).

#### 3.2.2 Vegetation greenness

**FIGURE 4:**
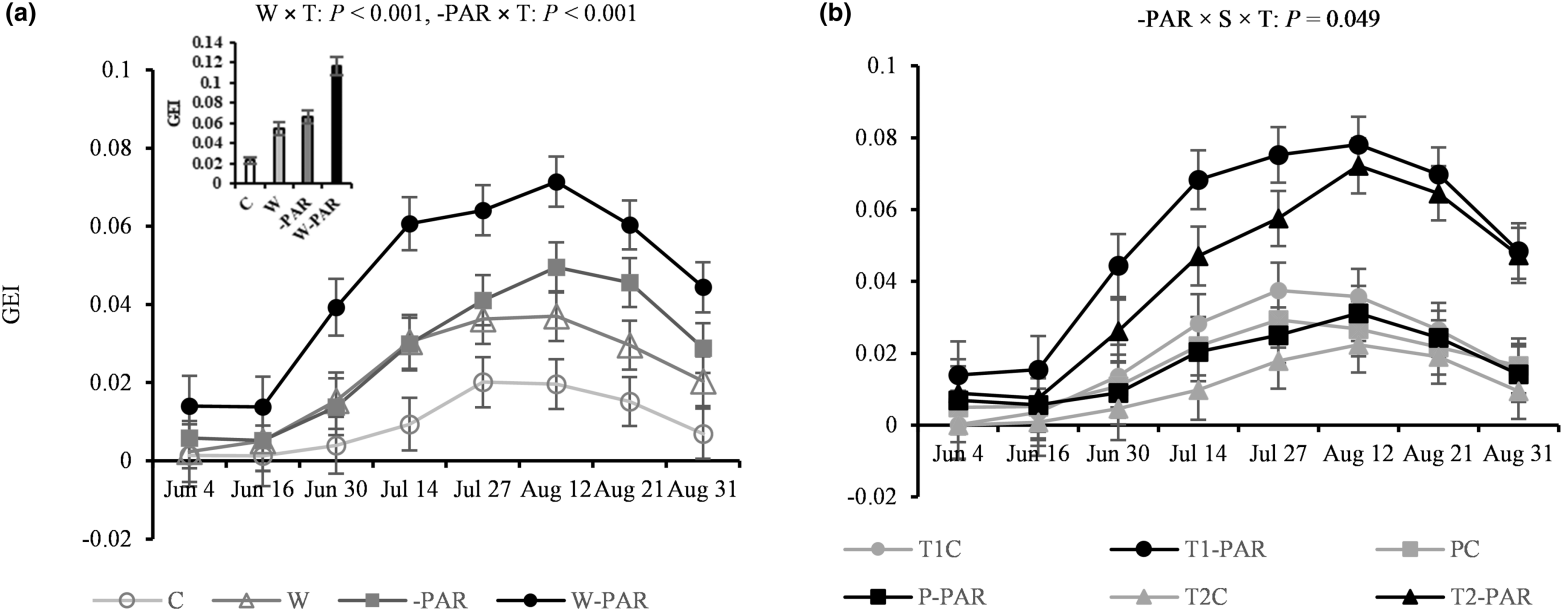
Greenness excess index (GEI) variability during 8 measurement campaigns in (a) control (C), warming (W), increased cloudiness (‐PAR), and warming + increased cloudiness (W‐PAR) treatments averaged across all sites (mean, *n* = 15 per treatment) and in (b) tundra1 (T1), tundra2 (T2) and palsa (P) sites under increased cloudiness treatments (mean, *n =* 5 per site, *n* = 30 per treatment). Bar chart inset (a) displays the mean GEI (±SE) across the growing season averaged across all sites per treatment. W × T = warming × time, ‐PAR × T = increased cloudiness × time, and ‐PAR × S × T = increased cloudiness × site × time interactions, T1C = T1 site in control, T1‐PAR = T1 site in increased cloudiness, T2C = T2 site in control, T2‐PAR = T2 site in increased cloudiness, PC = P site in control, P‐PAR = P site in increased cloudiness treatments.

Averaged across all sites, both warming and increased cloudiness increased nG and GEI from the end of June to the end of August (*p* < .001, Figure 4a; Figure S18a) resulting in an additive effect in the combined warming and increased cloudiness treatment (Figure 4a; Figure S18a,c). Unlike warming, the effect of increased cloudiness was dependent on site (*p* = .049, Figure 4b; Figure S18b). Both nG and GEI increased significantly from the end of June (for the T1 site) and mid‐July (for the T2 site), while the P site showed no change in nG and GEI throughout the season in response to increased cloudiness (Figure 4b; Figure S18b). nG and GEI increased gradually from the end of June, peaked on August 12, and subsequently decreased (Figure 4a; Figure S18a).

### 3.3 Leaf anatomy

Warming increased LMA of *V. myrtillus* and *R. chamaemorus* by 9.1% and 27.2%, respectively, (Tables 2 and 3) and increased the GT density on lower leaf surface of *V. myrtillus* by 38.0% (Table 2). Warming decreased *V. vitis‐idaea* spongy parenchyma thickness by 22.0%, while the intercellular space of spongy tissue also decreased under warming from 48.0% to 44.6% for *V. vitis‐idaea* and from 63.2% to 58.1% for *E. hermaphroditum* (Tables 2 and 3). Changes in the intercellular space of spongy tissues accounted for the observed changes in the total intercellular space under warming for *V. vitis‐idaea* (Table 3).

**TABLE 2:** Leaf anatomy variables of *Empetrum hermaphroditum* and *Vaccinium myrtillus* (mean ± SE) in control (C), warming (W), increased cloudiness (‐PAR), and warming + increased cloudiness (W‐PAR) treatments (*n* = 6–10 per treatment).

	Leaf anatomy				*p*‐value		
	C	W	‐PAR	W‐PAR	W	‐PAR	W × ‐PAR
*E. hermaphroditum*							
Shape change index	2.2 ± 0.1	2.0 ± 0.1	2.5 ± 0.1	2.3 ± 0.2	.203	**.040**	.754
Leaf thickness (μm)	262.3 ± 10.8	264.5 ± 7.2	263.1 ± 11.8	258.4 ± 5.1	.894	.773	.713
GT density on inner surface (#/mm^−1^)	3.3 ± 0.4	2.4 ± 0.4	2.8 ± 0.2	2.4 ± 0.1	.063	.492	.498
Stomata density on inner surface (#/mm^−1^)	3.0 ± 0.3	2.7 ± 0.4	2.1 ± 0.4	2.1 ± 0.2	.644	**.040**	.759
Outer epidermis thickness (μm)	22.9 ± 1.3	22.6 ± 0.8	20.2 ± 1.2	20.7 ± 2.0	.917	.123	.775
Inner epidermis thickness (μm)	8.9 ± 0.5	9.9 ± 0.5	9.4 ± 0.6	10.1 ± 0.5	.129	.461	.727
Palisade parenchyma thickness (μm)	88.1 ± 8.3	92.3 ± 5.9	80.4 ± 5.3	71.8 ± 2.5	.716	**.026**	.294
Spongy parenchyma thickness (μm)	101.7 ± 8.3	107.5 ± 6.9	96.7 ± 3.0	103.7 ± 7.0	.346	.508	.929
Intercellular space palisade (%)	14.8 ± 1.1	11.1 ± 1.7	12.5 ± 2.0	14.3 ± 1.8	.581	.802	.120
Intercellular space spongy (%)	63.2 ± 2.1	58.1 ± 1.6	63.0 ± 1.1	56.6 ± 3.5	**.020**	.716	.766
Intercellular space total (%)	39.7 ± 1.9	35.5 ± 0.5	39.0 ± 1.0	38.0 ± 2.7	.154	.598	.378
*V. myrtillus*							
Leaf thickness (μm)	144.0 ± 6.2	156.1 ± 5.3	145.8 ± 6.5	139.6 ± 5.9	.627	.234	.143
Leaf area (cm^−2^)	0.7 ± 0.5	0.7 ± 0.1	0.9 ± 0.1	0.8 ± 0.1	.213	**.037**	.365
Leaf dry mass per area (g m^−2^)	273.0 ± 2.3	297.9 ± 5.2	258.3 ± 12.6	268.2 ± 7.2	**.034**	**.008**	.346
GT density on upper surface (#/mm^−1^)	0.0 ± 0.0	0.1 ± 0.1	0.0 ± 0.0	0.0 ± 0.0	‐	‐	‐
GT density on lower surface (#/mm^−2^)	0.8 ± 0.2	1.1 ± 0.2	0.3 ± 0.1	1.1 ± 0.3	**.018**	.326	.284
Stomata density on upper surface (#/mm^−1^)	0.0 ± 0.0	3.4 ± 3.2	7.4 ± 7.1	0.0 ± 0.0	‐	‐	‐
Stomata density on lower surface (#/mm^−2^)	146.4 ± 10.1	165.6 ± 17.0	125.9 ± 15.4	148.9 ± 8.9	.126	.173	.887
Upper epidermis thickness (μm)	13.2 ± 0.4	12.8 ± 0.3	14.1 ± 0.3	12.5 ± 0.4	**.008**	.419	**.093**
Lower epidermis thickness (μm)	11.4 ± 0.2	11.3 ± 0.3	12.9 ± 0.2	11.3 ± 0.3	**.003**	**.008**	**.007**
Palisade parenchyma thickness (μm)	75.7 ± 3.7	87.0 ± 4.9	74.0 ± 5.3	67.4 ± 5.2	.632	**.039**	**.078**
Spongy parenchyma thickness (μm)	43.2 ± 2.5	45.5 ± 1.4	46.2 ± 2.1	47.2 ± 1.7	.418	.238	.756
Intercellular space palisade (%)	13.1 ± 0.8	11.5 ± 0.6	15.6 ± 1.7	13.5 ± 1.3	.128	.089	.884
Intercellular space spongy (%)	33.4 ± 1.6	33.9 ± 1.9	34.5 ± 1.6	35.7 ± 1.3	.614	.368	.830
Intercellular space total (%)	23.6 ± 1.7	21.9 ± 1.1	24.3 ± 1.7	26.7 ± 1.0	.815	.061	.156

*Note*: *p*‐values from Univariate ANOVA are shown and *p* < .1 emboldened.

Abbreviations: GT, glandular trichome; ‐, parameters tested by Kruskal–Wallis tests (*V. myrtillus*: GT density on upper surface *p* = .203, and stomata density on upper surface *p* = .166).

**TABLE 3:** Leaf anatomy variables of *Vaccinium vitis‐idaea* and *Rubus chamaemorus* (mean ± SE) in control (C), warming (W), increased cloudiness (‐PAR), and warming + increased cloudiness (W‐PAR) treatments (*n* = 4–9 per treatment).

	Leaf anatomy				*p*‐value		
	C	W	‐PAR	W‐PAR	W	‐PAR	W × ‐PAR
*V. vitis‐idaea*							
Leaf thickness (μm)	411.7 ± 17.4	327.0 ± 20.2	359.0 ± 16.8	369.2 ± 33.3	.127	.824	**.056**
Leaf area (cm^−2^)	0.8 ± 0.5	0.4 ± 0.1	0.7 ± 0.2	0.8 ± 0.1	‐	‐	‐
Leaf dry mass per area (g m^−2^)	284.5 ± 35.5	317.0 ± 49.0	276.1 ± 44.4	323.2 ± 50.1	.387	.980	.874
GT density on lower surface (#/mm^−2^)	1.3 ± 0.2	1.3 ± 0.3	1.1 ± 0.3	1.8 ± 0.6	.455	.979	.401
Stomata density on lower surface (#/mm^−2^)	371.7 ± 24.7	423.5 ± 23.5	302.5 ± 31.0	349.5 ± 19.9	.073	**.013**	.927
Upper epidermis thickness (μm)	16.8 ± 0.8	15.8 ± 1.2	18.2 ± 0.9	16.6 ± 0.5	.154	.233	.699
Lower epidermis thickness (μm)	12.3 ± 0.8	11.7 ± 0.4	12.0 ± 0.5	11.6 ± 0.2	.352	.686	.845
Palisade parenchyma thickness (μm)	183.6 ± 11.1	146.3 ± 11.1	151.0 ± 4.9	166.8 ± 24.1	.482	.690	.092
Spongy parenchyma thickness (μm)	192.9 ± 9.8	149.8 ± 11.5	176.9 ± 11.9	168.5 ± 9.7	**.027**	.900	.124
Intercellular space palisade (%)	15.0 ± 1.6	16.6 ± 1.4	20.3 ± 1.6	18.0 ± 1.5	.809	**.042**	.234
Intercellular space spongy (%)	48.0 ± 1.4	44.6 ± 1.4	52.3 ± 2.0	44.7 ± 1.6	**.003**	.194	.211
Intercellular space total (%)	31.3 ± 1.1	29.8 ± 1.3	37.8 ± 1.4	31.6 ± 1.9	**.018**	**.010**	.127
*R. chamaemorus*							
Leaf thickness (μm)	164.5 ± 14.5	197.3 ± 12.3	176.6 ± 12.1	158.0 ± 15.0	.609	.337	.082
Leaf area (cm^−2^)	3.3 ± 0.4	4.2 ± 0.7	4.4 ± 1.1	6.3 ± 1.1	.146	.096	.592
Leaf dry mass per area (g m^−2^)	95.1 ± 34.6	121.0 ± 25.5	79.9 ± 37.8	224.9 ± 100.2	**.045**	.803	.348
GT density on upper surface (#/mm^−2^)	1.5 ± 0.5	1.3 ± 0.9	1.3 ± 0.5	1.6 ± 1.0	.829	.933	.796
GT density on lower surface (#/mm^−2^)	4.6 ± 2.2	3.5 ± 1.9	5.5 ± 1.9	6.4 ± 3.3	.748	.617	.960
Stomata density on lower surface (#/mm^−2^)	223.5 ± 21.0	192.8 ± 33.5	180.9 ± 9.8	197.0 ± 39.8	.803	.513	.427
Hair density on upper surface (#/mm^−1^)	0.0 ± 0.1	0.0 ± 0.0	1.9 ± 3.6	0.0 ± 0.1	‐	‐	‐
Upper epidermis thickness (μm)	18.2 ± 0.7	18.5 ± 0.8	17.7 ± 0.7	18.7 ± 1.0	.403	.903	.631
Lower epidermis thickness (μm)	19.1 ± 0.8	18.7 ± 0.6	17.6 ± 0.4	17.9 ± 0.8	.930	.101	.625
Palisade parenchyma thickness (μm)	71.8 ± 6.1	87.3 ± 13.7	61.8 ± 5.3	69.9 ± 6.5	.195	.135	.676
Spongy parenchyma thickness (μm)	59.9 ± 8.4	61.5 ± 7.8	55.6 ± 7.3	51.1 ± 3.7	.837	.315	.667
Intercellular space palisade (%)	23.7 ± 3.1	23.4 ± 0.9	25.1 ± 4.9	25.3 ± 1.9	.977	.615	.931
Intercellular space spongy (%)	56.4 ± 3.6	55.5 ± 8.7	54.1 ± 4.9	53.9 ± 4.6	.925	.740	.953
Intercellular space total (%)	38.6 ± 3.0	36.9 ± 4.1	37.9 ± 4.3	39.0 ± 3.9	.932	.862	.723

*Note*: *p*‐values from Univariate ANOVA are shown and *p* < .1 emboldened.

Abbreviations: GT, glandular trichome; ‐, indicates parameters tested by Kruskal–Wallis tests (*V. vitis‐idaea*: leaf area *p* = .077; *R. chamaemorus*: hair density on upper surface *p* = .366).

For *E. hermaphroditum*, increased cloudiness made the needle‐like curled leaves (Figure 1a) broader, but flatter (significantly higher shape change index, Table 2) and decreased the stomata density (per inner leaf surface length, Figure 1a) and palisade parenchyma thickness by 30% and 9.0%, respectively (Table 2). For *V. myrtillus*, increased cloudiness increased the LA by 28.6% and decreased the LMA by 5.4% (Table 2). Increased cloudiness decreased the stomata density (present only on the lower leaf surface) of *V. vitis‐idaea* by 19.0% (Table 3) and increased the intercellular space of palisade tissue from 15.0% to 20.3% (Table 3). Changes in the intercellular space of palisade tissues accounted for the observed changes in the total intercellular space under increased cloudiness for *V. vitis‐idaea* (Table 3).

There was a significant warming × increased cloudiness (W × ‐PAR) interaction on the upper and lower epidermis, and the palisade parenchyma thicknesses of *V. myrtillus* (Table 2). Warming decreased the upper and lower epidermis thicknesses with increased cloudiness (Table 2; Table S8). Increased cloudiness, by contrast, increased the lower epidermis thickness without warming (Table 2; Table S8). Increased cloudiness decreased the palisade parenchyma thickness with warming (Table 2; Table S8). There was also a W × ‐PAR interaction on the leaf thickness of *V. vitis‐idaea* (Table 3). Warming decreased the leaf thickness without increased cloudiness (Table 3; Table S8). There were no significant interactions between warming and increased cloudiness on leaf anatomy of *E. hermaphroditum* and *R. chamaemorus*.

### 3.4 Leaf chlorophyll, C, and N content

**FIGURE 5:**
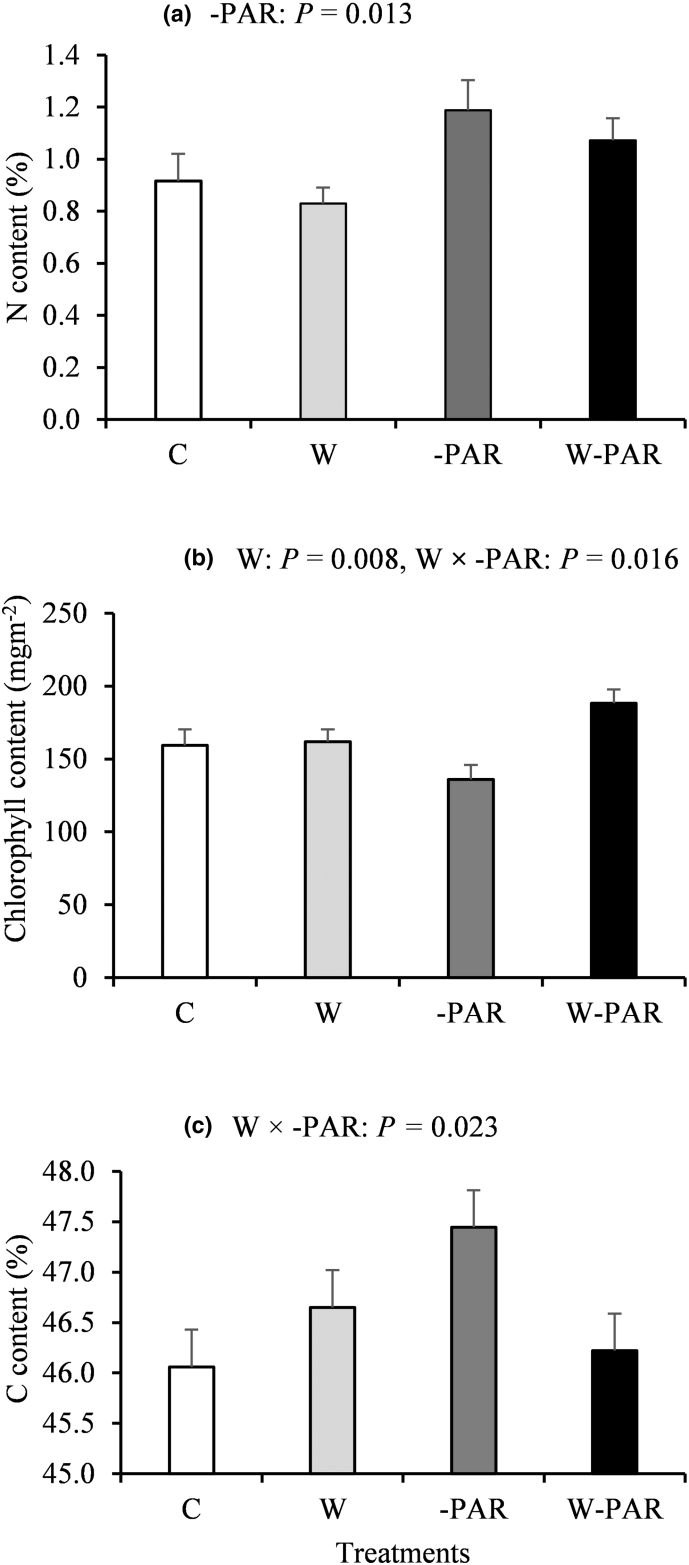
(a) Leaf N and (b) chlorophyll content (mean ± SE, *n* = 6–11) of *Empetrum hermaphroditum* and (c) leaf carbon content (mean ± SE, *n* = 6–11) of *V. myrtillus* in control (C), warming (W), increased cloudiness (‐PAR), and warming + increased cloudiness (W‐PAR) treatments. *p*‐values <.05 for main and interaction effects of W and ‐PAR from univariate ANOVA are shown.

Increased cloudiness increased leaf N of *E. hermaphroditum* (Figure 5a). Warming as a factorial response significantly increased leaf chlorophyll of *E. hermaphroditum* and this was mainly due to a strong interaction effect when the warming and increased cloudiness treatments were combined, which further enhanced leaf chlorophyll of *E. hermaphroditum* (Figure 5b; Table S8). Leaf C of *V. myrtillus* increased in response to increased cloudiness without warming and decreased when warming and increased cloudiness acted together (W × ‐PAR interaction, Figure 5c; Table S8).

Treatment effects on leaf chlorophyll content of *V. myrtillus*, *V. vitis‐idaea* and *R. chamaemorus* were not significant (Figure S19a–c). There were no treatment effects on leaf C and N content of *E. hermaphroditum* and *V. myrtillus*, respectively (Figure S20a,b).

There were no correlations between leaf C and N content and leaf anatomy variables of the abundant main vascular plant species *E. hermaphroditum* and *V. myrtillus* as well as between soil C and N content, and leaf anatomy and total biomass variables of all the main vascular plant species (data not shown).

## 4 DISCUSSION

### 4.1 Effects of warming

As expected, warming enhanced mesocosm vegetation greenness as greenness index (nG) and greenness excess index (GEI) values increased during the growing season in response to warming. Despite the increase in greenness, vegetation cover and biomass showed no change or decreased in response to warming. Such asynchronous change in vegetation under warming is contrary to our hypotheses but supports previous findings showing that vegetation greenness may not be proportionally translated into productivity and ecosystem functioning (Ding et al., 2020; Zhao et al., 2022). Contrary to our hypotheses, warming also did not affect the leaf N and C of the dominant species. However, in line with our hypotheses, warming increased the biomass and leaf chlorophyll of one of the dominant species (*E. hermaphroditum*) indicating increased leaf photosynthesis. This may have contributed to the observed changes in nG and GEI and thus vegetation greenness at the ecosystem level. This is because greenness ratios derived from true‐color digital images have a strong positive correlation with chlorophyll content (Adamsen et al., 1999) and green biomass (Migliavacca et al., 2011) and hence can be used to monitor changes in biomass and productivity (Beamish et al., 2016). The soil properties of the T2 site, such as slightly lower OM content, higher bulk density, and lower WHC compared to the other sites, could perhaps explain the observed decrease in the cover of deciduous shrubs in response to warming. These soil characteristics can negatively affect water and nutrient availability, microbial activity, biochemical processes, photosynthesis, and may act as a potential limitation of shrub growth responses to warming (Gamm et al., 2018; Myers‐Smith et al., 2015; Nadelhoffer et al., 1991). The lack of significant alterations in the cover and biomass of other plant species or functional groups reflects notable variation across species in the scale of their sensitivity to warming or the abovementioned factors that could limit species growth responses to warming.

Within a given species, LMA usually has a strong connection with photosynthesis, since thicker leaves with more palisade parenchyma tissue tend to have both higher LMA and photosynthetic capacity (Manishimwe et al., 2022; Poorter et al., 2009). Therefore, the higher LMA under warming for *V. myrtillus* and *R. chamaemorus* indicates improved photosynthetic capacity in these deciduous species. Higher density of glandular trichomes on the lower leaf surface of *V. myrtillus* under warming will enhance the plants' defense capacity against abiotic and biotic stressors such as heat and water stress (Schollert et al., 2017; Thitz et al., 2017), herbivores (Glas et al., 2012; Valkama et al., 2004), and microbial infections (Muravnik & Shavarda, 2012).

Decreased intercellular space of the spongy tissue in response to warming for the evergreen species *E. hermaphroditum* and *V. vitis‐idaea* indicates structural acclimation limiting mesophyll conductance and diffusion of water vapor in the intercellular space thereby enhancing the water‐use efficiency. The evergreen species showed similar leaf anatomical acclimation strategies, but different from that of the deciduous species, in response to warming. These anatomical acclimation strategies will improve growth and survival, and thus enhance cover of the deciduous and evergreen species in the long‐term under future temperature rise.

### 4.2 Effects of increased cloudiness

Surprisingly, nG and GEI values increased during the growing season in response to increased cloudiness in the tundra sites, indicating increased greenness. This finding indicates that species differences, for example, those of deciduous shrubs, graminoids, forbs or mosses dominant in tundra, and their ability to respond to increased cloudiness are driving nG and GEI increases. Therefore, the presence and inherently high cover of these growth forms had a greater influence on nG and GEI values in the tundra sites compared to the more sparsely vegetated palsa site where nG and GEI pattern was weakest, and litter and bare soil were extensive. Despite the increase in greenness, the cover of evergreen shrubs in all sites and of deciduous shrubs and lichens in the T2 site decreased in response to increased cloudiness with no change in the biomass or cover of other functional groups of plant species. This further supports the decoupled relationship that may exist between greenness and productivity. The decreased deciduous shrub cover for the T2 site could also be due to restricted plant growth associated with the slightly lower OM content, higher bulk density, and lower WHC of the T2 site compared to the other sites as was observed under warming. The lack of responses of other plant functional groups in the T2 site further highlights variations across species in their responses to the abovementioned soil related properties that could limit growth and distribution. The high cover of mosses, irrespective of sites, in response to increased cloudiness reflects physiological adaptation and ability of these growth forms to thrive in low light intensities which are not favorable for the vascular plant species.

Plants growing under shade conditions tend to produce thinner leaves with greater LA and lower LMA compared to the same species growing under higher light intensity (Gurevitch et al., 2006; Lichtenthaler et al., 1981; Terashima et al., 2011). We found increased LA and decreased LMA for *V. myrtillus* in response to increased cloudiness, fitting the shade‐leaf habit of reducing leaf mass as a means to increase area for light interception (Baird et al., 2017), and the increased LA may have contributed to the enhanced vegetation greenness. However, the lower epidermis was also thicker indicating a structural acclimation which will enhance water‐use efficiency (Schollert et al., 2017). These leaf anatomical acclimations will enhance tolerance against stress, and thus minimize growth and distribution losses for the deciduous shrub species under increased cloudiness.

The evergreen species showed a decrease in stomata density on the inner/lower surface in response to increased cloudiness, indicative of lower stomata conductance and reduced stomata transpiration. Decreased thickness of the palisade parenchyma under increased cloudiness for *E. hermaphroditum* can be suppressive for photosynthetic efficiency as the thinner palisade parenchyma can be indicative of less sites for photosynthesis (Hartikainen et al., 2009; Schollert et al., 2015). Similarly, the observed increase in palisade intercellular space and associated increase in total intercellular space for *V. vitis‐idaea* under increased cloudiness is indicative of a less tightly packed palisade parenchyma providing less sites for photosynthesis (Hartikainen et al., 2009; Schollert et al., 2015), hence lower photosynthetic potential. Therefore, the evergreen shrubs will develop lower photosynthetic efficiency under future increased cloudiness conditions. However, broader leaves, indicated by higher leaf shape change index, and thinner palisade for *E. hermaphroditum*, and higher palisade and total intercellular space for *V. vitis‐idaea* will provide an opportunity for higher light harvesting, internal scattering and absorbance, thereby enhancing photosynthesis and minimizing growth and distribution losses. Increased N content in leaves of *E. hermaphroditum* in response to increased cloudiness indicates a strategy by this species to compensate for lower photosynthetic capacity through increased resource allocation to leaves (Hansen et al., 2006; Iason & Hester, 1993; Michelsen et al., 1996). Together with broader leaves, the increased N content may also have contributed to the enhanced vegetation greenness under increased cloudiness. Therefore, leaf anatomical alterations under increased cloudiness for the evergreen species will impair their photosynthetic potential. However, both evergreen and deciduous species will enhance their tolerance against stress and minimize growth and distribution losses associated with increased cloudiness. Furthermore, the lack of significant alterations in trichome densities and leaf biochemical traits of the dominant species, except for increased leaf N of *E. hermaphroditum*, is contrary to our hypotheses but also reflects variation in the responses of plant species to increased cloudiness.

### 4.3 Effects of combined warming and increased cloudiness

Across all sites, the combined warming and increased cloudiness treatment produced an additive effect on vegetation greenness. The graminoids and forbs could thrive and grow taller and larger as their cover increased more under combined warming and increased cloudiness than under warming or increased cloudiness alone, in the site with high abundance of these species (T1 site). This may have contributed to the enhanced vegetation greenness. Increased chlorophyll concentration in leaves of *E. hermaphroditum*, indicating an enhancement of their photosynthetic capacity and productivity, may also have contributed to enhanced greenness of the vegetation cover (Adamsen et al., 1999) when warming and increased cloudiness were combined.

Warming and increased cloudiness modified the effects of each other towards a thinner lower and upper leaf epidermis and palisade parenchyma for *V. myrtillus*, which have important implications for photosynthesis and protection, hence survival and growth. As the tightly packed palisade parenchyma beneath the top epidermis has a high potential for photosynthesis (Monson & Baldocchi, 2014), a leaf with thinner palisade parenchyma, on the other hand, will offer less sites for photosynthesis thereby reducing photosynthetic potential coupled with limited availability of C especially under reduced light (increased cloudiness). Thicker leaf and epidermis is beneficial for plants under water deficit conditions (Larcher, 2003; Schollert et al., 2017), while thinner epidermis, on the other hand, facilitates water loss through increased evapotranspiration. Such anatomical acclimations impairing photosynthesis and water relations will impair survival and growth and decrease the cover of the deciduous shrubs in the long‐term. Increased leaf C concentration under increased cloudiness without warming for *V. myrtillus* reflects a pattern of resource allocation to leaves to compensate for lower photosynthetic capacity under reduced light conditions. These leaf anatomical and chemical acclimations further indicate that the deciduous species appear most sensitive and vulnerable, compared to the evergreen species, when facing future warmer increased cloudiness conditions. This is compounded by the generally thinner leaves, shorter photosynthetic activity season, and higher herbivore palatability of the deciduous species (Chapin et al., 1996).

It has been shown that leaf and soil nutrients, like climate, are key factors that regulate leaf morphological and anatomical traits (Tian et al., 2016), and aboveground biomass (Chapin et al., 1995). However, we found no correlations between leaf nutrient concentrations and leaf anatomical traits of the abundant vascular plant species *E. hermaphroditum* and *V. myrtillus*. Likewise, no correlations were observed between soil nutrients and leaf anatomical traits and aboveground biomass of any of the main vascular plant species. Hence, the observed changes in leaf anatomy and biomass were likely due to the direct effect of the treatments rather than an indirect effect of changes in foliar or soil nutrient content.

### 4.4 Implications and future directions

Collectively, it appears that warming coupled with increased cloud cover in high latitude regions will result in a range of ecosystem and plant species changes, including changes in vegetation greenness and cover, and leaf anatomy and biochemistry. These changes may have consequences for carbon gain and primary productivity, and abiotic and biotic stress tolerance. Our findings indicates that despite increases in vegetation greenness, warming or increased cloudiness will decrease the cover of shrubs, while the cover of graminoids and forbs will increase in response to combined warming and increased cloudiness. Our findings support the decoupled change that may occur between vegetation greenness and cover or biomass. Other site‐specific attributes that will directly or indirectly alter ecosystem processes, the abundance of species growth forms, and leaf anatomical and biochemical trait responses to warming and increased cloudiness will, in addition, be driving factors in shaping subarctic vegetation species compositional distribution, canopy structure, productivity, and ecosystem functioning. Our observed responses of Subarctic vegetation to warming or increased cloudiness after 2 years of rigorously controlled treatments are consistent with results from long‐term field experiments experimenting with these environmental factors (Gamm et al., 2018; Graglia et al., 1997; Michelsen et al., 1996; Myers‐Smith et al., 2015; Ndah et al., 2022; Schollert et al., 2015, 2017), although mostly with a lower level of warming than that achieved with our experimental setup simulating 4°C warming and hence more realistic changes to longer term temperature conditions in the subarctic. Therefore, our observed responses to combined warming and increased cloudiness after two growing seasons could predict responses in the long‐term.

Our study involved climate chambers which allowed the simulation of combined effects of warming and increased cloudiness under highly controlled environmental conditions over two growing seasons. The next step in unraveling the effects of climate change on subarctic vegetation composition and leaf traits should be geared towards investigating the combined effects of warming and increased cloudiness over a longer time span in field conditions. Moreover, simulating cloud radiative forcing in chamber experiments like in our study is a major challenge. Potential future work including projections of cloud formation through more sophisticated modeling techniques that include changes in land‐surface physical characteristics, such as albedo which controls land‐atmosphere interactions, can be beneficial to verify our results and strengthen future predictions of climate change effects on subarctic vegetation.

## CONFLICT OF INTEREST STATEMENT

Authors declare no conflict of interest.

## Supporting information


Data S1:

## Data Availability

The data that support the findings of this study will be made openly available in Figshare upon acceptance.
