# Supplementary material for: Acclimation of subarctic vegetation to warming and increased cloudiness
Source: Plant Environ Interact. 2023 Nov 28;5(1):e10130. doi: 10.1002/pei3.10130 (PMC10840376; doi:10.1002/pei3.10130)
Supplement: Supplementary file 1 — Data S1: [file PEI3-5-e10130-s001.docx]

**Supporting information**

**for**

**Acclimation of subarctic vegetation to warming and increased cloudiness**

Flobert A. Ndah^*^, Marja Maljanen, Anne Kasurinen, Riikka Rinnan, Anders Michelsen, Titta Kotilainen, Minna Kivimäenpää

^*^Corresponding author: Flobert A. Ndah, Department of Environmental and Biological Sciences, University of Eastern Finland, P.O Box 1627, 70211, Kuopio, Finland. Email: [flobert.ndah@uef.fi](mailto:flobert.ndah@uef.fi)

Table S1. Results of simple main effect (SME) tests for principal component (PC) score data on plant functional groups. Score data (PC1 and PC2) were obtained from principal component analysis i.e., tested variables represent scores for % coverage of the plant functional groups; deciduous shrubs, mosses, lichens, evergreen shrubs, litter + bare soil (PC1) and graminoids, forbs, evergreen shrubs, and lichens (PC2) prior to exposure to experimental treatments in 2020 (i.e., analysis was done on mesocosms after their collection in late growing season 2019 and before their exposure to experimental treatments in the following year 2020). Therefore, significant site differences here indicate variations that appeared naturally between the different studied sites. Abbreviations: S = site, T1 = tundra1 site, P = palsa site, T2 = tundra2 site. Only statistically significant (*P*-value ≤ 0.05) are shown (*n* = 20 per site).

| Year | Plant functional groups | Tested variable | main effects | SME comparison | SME test *P*-value |
| --- | --- | --- | --- | --- | --- |
| 2019 |  |  |  |  |  |
|  | Deciduous shrubs, mosses, lichens, evergreen shrubs, and litter + bare soil | PC1 | S | T1 vs T2 | 0.040 |
|  |  |  |  | T1 vs P | < 0.001 |
|  |  |  |  | T2 vs P | < 0.001 |
|  | graminoids, forbs, evergreen shrubs, and lichens | PC2 | S | T1 vs T2 | < 0.001 |
|  |  |  |  | T1 vs P | < 0.001 |

Table S2. Photosynthetically active radiation (PAR) and spectral composition of simulated irradiances for Vassijaure and measured irradiances from Valoya luminaires in the climate chambers. Irradiance measurements in the climate chambers were performed using a Portable High-Accuracy UV-Visible Spectroradiometer (OL 756, Optronic laboratories, Inc., Orlando, Florida, USA). Both the irradiance simulations and the measurements of irradiance from the luminaires in the wavelength range from 280 nm to 800 nm were processed in R (R Core Team, 2017), using the photobiology packages developed for spectral analysis (Aphalo 2015). UVB radiation and UVA radiation are defined according to ISO (2007). In plant science comparison of light sources and assessment of the relative contributions of different wavebands beyond the UV wavebands is commonly done by dividing them into 100-nm increments (Both et al. 2017). Thus, PAR = 400-700 nm, blue = 400-500 nm, green = 500-600 nm, red = 600-700 nm, and far-red = 700-800 nm.

| Percentage out of total irradiance 280-800 nm | | | | | | | |
| --- | --- | --- | --- | --- | --- | --- | --- |
|  | PAR (µmol m^-2^s^-1^ ) 400-700 | UVB 280-315 | UVA 315-400 | Blue 400-500 | Green 500-600 | Red 600-700 | Far-red 700-800 |
| Vassijaure, 22 June 2018 (noon), no clouds | 1470 | 0.1 | 5.9 | 19.9 | 24.8 | 25.7 | 23.6 |
| Vassijaure, 22 June 2018 (noon), cloud optical depth ten | 784 | 0.1 | 6.3 | 19.9 | 24.6 | 25.2 | 24.0 |
| Climate chamber without the mesh | 880 | - | 0.2 | 15.3 | 27.1 | 46.6 | 10.7 |
| Climate chamber with the mesh | 767 | - | 0.2 | 15.1 | 27.2 | 46.8 | 10.8 |

Table S3. Vegetation coverage (%, mean ± S.E, *n* = 5 per site, *n* = 15 per treatment) of plant species functional groups, individual plant species, and litter + bare soil in tundra1 (T1), tundra2 (T2), and palsa (P) sites, and in control (C), warming (W), increased cloudiness (-PAR) and warming + increased cloudiness (W-PAR) treatments in 2020.

| **Plant species functional groups** | **T1** | **P** | **T2** | **C** | **W** | **-PAR** | **W-PAR** |
| --- | --- | --- | --- | --- | --- | --- | --- |
| Evergreen shrubs | 10.0 ± 2.4 | 23.8 ± 4.3 | 13.5 ± 2.6 | 23.1 ± 5.2 | 18.0 ± 4.2 | 11.3 ± 3.0 | 10.5 ± 2.2 |
| Deciduous shrubs | 8.7 ± 1.8 | 0.8 ± 0.4 | 9.7 ± 1.9 | 12.0 ± 3.0 | 6.4 ± 1.8 | 3.7 ± 1.0 | 3.3 ± 1.0 |
| Graminoids | 2.3 ± 0.7 | 0.0 ± 0.0 | 0.5 ± 0.2 | 1.1 ± 0.7 | 0.5 ± 0.2 | 0.7 ± 0.5 | 1.3 ± 0.7 |
| Forbs | 5.5 ± 1.2 | 1.8 ± 0.5 | 0.0 ± 0.0 | 2.4 ± 1.0 | 3.4 ± 1.6 | 1.5 ± 0.5 | 2.4 ± 1.0 |
| Mosses | 85.7 ± 5.3 | 10.2 ± 4.8 | 92.5 ± 8.2 | 52.9 ± 9.1 | 59.9 ± 11.2 | 83.8 ± 14.5 | 54.5 ± 12.2 |
| Lichens | 0.5 ± 0.2 | 1.4 ± 0.6 | 11.9 ± 3.6 | 9.7 ± 4.9 | 4.7 ± 1.9 | 1.8 ± 0.7 | 2.1 ± 0.7 |
| Litter + bare soil | 4.0 ± 3.7 | 7.8 ± 4.8 | 0.1 ± 0.1 | 14.6 ± 7.6 | 0.1 ± 0.1 | 0.6 ± 0.3 | 0.4 ± 0.2 |
| **Plant species** |  |  |  |  |  |  |  |
| **Evergreen shrubs** |  |  |  |  |  |  |  |
| *Empetrum hermaphroditum* | 7.8 ± 2.4 | 13.7 ± 3.8 | 10.3 ± 1.8 | 17.1 ± 4.8 | 12.5 ± 2.8 | 6.5 ± 1.9 | 6.5 ± 1.7 |
| *Vaccinium vitis-idaea* | 0.7 ± 0.3 | 7.9 ± 1.9 | 1.4 ± 0.2 | 3.6 ± 1.4 | 3.3 ± 1.3 | 3.7 ± 2.3 | 2.9 ± 1.0 |
| *Phyllodoce caerulea* | 1.9 ± 0.6 | 0.0 ± 0.0 | 0.0 ± 0.0 | 1.3 ± 0.7 | 0.3 ± 0.3 | 0.3 ± 0.3 | 0.6 ± 0.4 |
| *Andromeda polifolia* | 0.0 ± 0.0 | 2.3 ± 0.8 | 0.1 ± 0.1 | 1.1 ± 0.6 | 1.1 ± 0.7 | 0.8 ± 0.7 | 0.1 ± 0.1 |
| *Kalmia procumbens* | 0.0 ± 0.0 | 0.0 ± 0.0 | 1.8 ± 1.3 | 0.0 ± 0.0 | 1.7 ± 1.7 | 0.0 ± 0.0 | 0.1 ± 0.1 |
| **Deciduous shrubs** |  |  |  |  |  |  |  |
| *Vaccinium myrtillus* | 8.6 ± 1.8 | 0.7 ± 0.4 | 9.4 ± 1.9 | 11.5 ± 3.0 | 6.4 ± 1.8 | 3.7 ± 0.9 | 3.2 ± 1.0 |
| *Vaccinium uliginosum* | 0.0 ± 0.0 | 0.0 ± 0.0 | 0.3 ± 0.1 | 0.2 ± 0.1 | 0.0 ± 0.0 | 0.0 ± 0.0 | 0.1 ± 0.1 |
| *Betula nana* | 0.1 ± 0.1 | 0.1 ± 0.1 | 0.1 ± 0.1 | 0.3 ± 0.2 | 0.0 ± 0.0 | 0.1 ± 0.1 | 0.0 ± 0.0 |
| **Graminoids** |  |  |  |  |  |  |  |
| *Deschampsia flexuosa* | 2.3 ± 0.7 | 0.0 ± 0.0 | 0.5 ± 0.2 | 1.1 ± 0.7 | 0.5 ± 0.2 | 0.7 ± 0.5 | 1.3 ± 0.7 |
| **Forbs** |  |  |  |  |  |  |  |
| *Rubus chamaemorus* | 2.0 ± 0.5 | 1.8 ± 0.5 | 0.0 ± 0.0 | 1.2 ± 0.7 | 1.7 ± 0.6 | 0.9 ± 0.4 | 1.1 ± 0.4 |
| *Conus suecica* | 3.5 ± 1.1 | 0.0 ± 0.0 | 0.0 ± 0.0 | 1.1 ± 0.7 | 1.7 ± 1.3 | 0.5 ± 0.4 | 1.3 ± 0.5 |
| *Pedicularis lapponica* | 0.1 ± 0.1 | 0.0 ± 0.0 | 0.0 ± 0.0 | 0.1 ± 0.1 | 0.0 ± 0.0 | 0.1 ± 0.1 | 0.0 ± 0.0 |
| *Cerastium alpinum* | 0.0 ± 0.0 | 0.1 ± 0.1 | 0.0 ± 0.0 | 0.1 ± 0.1 | 0.0 ± 0.0 | 0.0 ± 0.0 | 0.0 ± 0.0 |
| **Mosses** |  |  |  |  |  |  |  |
| *Pleurozium schreberi* | 75.8 ± 5.7 | 0.0 ± 0.0 | 65.3 ± 8.1 | 40.7 ± 9.8 | 48.3 ± 11.5 | 57.3 ± 11.2 | 41.7 ± 11.6 |
| *Polytrichum commune* | 0.2 ± 0.1 | 1.7 ± 1.0 | 0.3 ± 0.2 | 1.3 ± 1.3 | 0.5 ± 0.2 | 0.5 ± 0.2 | 0.6 ± 0.3 |
| *Dicranum* spp. | 7.2 ± 2.0 | 8.5 ± 4.7 | 27.0 ± 6.5 | 10.9 ± 4.4 | 7.6 ± 3.2 | 26.0 ± 8.5 | 12.3 ± 5.5 |
| *Hylocomium splendens* | 2.6 ± 2.5 | 0.0 ± 0.0 | 0.0 ± 0.0 | 0.0 ± 0.0 | 3.5 ± 3.3 | 0.0 ± 0.0 | 0.0 ± 0.0 |
| **Lichens** |  |  |  |  |  |  |  |
| *Nephroma arcticum* | 0.1 ± 0.1 | 0.0 ± 0.0 | 4.8 ± 1.8 | 2.8 ± 2.0 | 3.2 ± 1.7 | 0.3 ± 0.2 | 0.1 ± 0.1 |
| *Cladonia* spp. | 0.1 ± 0.1 | 1.2 ± 0.5 | 2.2 ± 0.6 | 1.5 ± 0.8 | 0.6 ± 0.3 | 0.8 ± 0.4 | 1.7 ± 0.7 |
| *Cladonia arbuscular* | 0.0 ± 0.0 | 0.2 ± 0.1 | 1.2 ± 0.5 | 1.1 ± 0.7 | 0.6 ± 0.2 | 0.0 ± 0.0 | 0.1 ± 0.1 |
| *Stereocaulon paschale* | 0.0 ± 0.0 | 0.1 ± 0.1 | 3.6 ± 2.5 | 3.7 ± 3.3 | 0.3 ± 0.1 | 0.7 ± 0.4 | 0.1 ± 0.1 |
| *Thamnolia vermicularis* | 0.3 ± 0.2 | 0.0 ± 0.0 | 0.3 ± 0.1 | 0.6 ± 0.3 | 0.0 ± 0.0 | 0.0 ± 0.0 | 0.1 ± 0.1 |
| **Litter + bare soil** | 4.0 ± 3.7 | 7.8 ± 4.8 | 0.1 ± 0.1 | 14.6 ± 7.6 | 0.1 ± 0.1 | 0.6 ± 0.4 | 0.4 ± 0.2 |

Table S4. Vegetation coverage (%, mean ± S.E, *n* = 5 per site, *n* = 15 per treatment) of plant species functional groups, individual plant species, standing dead, litter, and bare soil in tundra1 (T1), palsa (P), and tundra2 (T2) sites in control (C), warming (W), increased cloudiness (-PAR) and warming + increased cloudiness (W-PAR) treatments in 2021. Standing dead = dead vegetation with root connections.

| **Plant species functional groups** | **T1** | **P** | **T2** | **C** | **W** | **-PAR** | **W-PAR** |
| --- | --- | --- | --- | --- | --- | --- | --- |
| Evergreen shrubs | 20.5 ± 4.3 | 35.1 ± 6.6 | 30.0 ± 4.2 | 24.6 ± 6.0 | 27.8 ± 6.8 | 27.8 ± 5.2 | 34.0 ± 6.6 |
| Deciduous shrubs | 22.7 ± 4.1 | 3.7 ± 1.4 | 30.2 ± 3.1 | 15.5 ± 3.5 | 19.0 ± 5.2 | 19.4 ± 4.7 | 21.5 ± 4.8 |
| Graminoids | 18.2 ± 6.1 | 0.0 ± 0.0 | 2.8 ± 1.6 | 5.7 ± 3.7 | 6.0 ± 3.8 | 4.3 ± 3.9 | 12.1 ± 6.6 |
| Forbs | 14.8 ± 3.0 | 2.6 ± 0.8 | 0.0 ± 0.0 | 5.1 ± 2.3 | 5.4 ± 2.6 | 2.6 ± 1.3 | 10.0 ± 3.7 |
| Mosses | 84.4 ± 5.6 | 16.1 ± 5.1 | 66.3 ± 4.3 | 61.1 ± 8.6 | 51.0 ± 10.3 | 59.7 ± 10.3 | 50.6 ± 9.1 |
| Lichens | 2.6 ± 1.4 | 5.1 ± 1.8 | 50.2 ± 5.2 | 24.9 ± 8.8 | 16.0 ± 5.1 | 14.4 ± 6.2 | 21.9 ± 6.9 |
| Vascular cryptograms | 0.0 ± 0.0 | 0.0 ± 0.0 | 1.0 ± 1.0 | 0.0 ± 0.0 | 0.0 ± 0.0 | 1.4 ± 1.4 | 0.0 ± 0.0 |
| Standing dead | 14.8 ± 2.6 | 29.3 ± 3.4 | 17.9 ± 2.8 | 24.2 ± 4.5 | 19.9 ± 4.0 | 20.7 ± 3.0 | 17.9 ± 3.4 |
| Litter | 2.7 ± 1.1 | 20.9 ± 2.4 | 0.6 ± 0.3 | 6.3 ±2.5 | 7.6 ± 3.0 | 10.6 ± 3.8 | 7.9 ± 2.7 |
| Bare soil | 0.3 ± 0.3 | 10.7 ± 2.0 | 0.0 ± 0.0 | 5.0 ± 2.1 | 4.2 ± 2.2 | 1.9 ± 1.0 | 3.6 ± 2.0 |
| **Plant species** |  |  |  |  |  |  |  |
| **Evergreen shrubs** |  |  |  |  |  |  |  |
| *Empetrum nigrum* | 13.2 ± 3.7 | 24.2 ± 4.9 | 26.8 ± 3.8 | 18.3 ± 4.4 | 22.1 ± 4.7 | 19.4 ± 4.5 | 25.7 ± 6.3 |
| *Vaccinium vitis-idaea* | 1.4 ± 0.6 | 6.0 ± 1.7 | 2.1 ± 0.6 | 1.7 ± 0.9 | 2.6 ± 1.4 | 5.0 ± 1.6 | 3.3 ± 1.4 |
| *Phyllodoce caerulea* | 5.7 ± 2.2 | 0.0 ± 0.0 | 0.2 ± 0.2 | 2.1 ± 1.3 | 0.3 ± 0.3 | 1.7 ± 1.7 | 3.9 ± 2.4 |
| *Andromeda polifolia* | 0.2 ± 0.2 | 4.9 ± 2.0 | 0.1 ± 0.1 | 2.5 ± 1.9 | 1.8 ± 1.5 | 1.7 ± 1.5 | 1.0 ± 0.7 |
| *Kalmia procumbens* | 0.0 ± 0.0 | 0.0 ± 0.0 | 0.8 ± 0.7 | 0.0 ± 0.0 | 1.0 ± 1.0 | 0.0 ± 0.0 | 0.1 ± 0.1 |
| **Deciduous shrubs** |  |  |  |  |  |  |  |
| *Vaccinium myrtillus* | 20.2 ± 3.4 | 1.9 ± 1.2 | 29.0 ± 2.8 | 13.8 ± 3.8 | 17.6 ± 4.6 | 18.8 ± 4.8 | 17.9 ± 3.9 |
| *Vaccinium uliginosum* | 1.6 ± 0.9 | 1.1 ± 0.6 | 1.3 ± 0.8 | 1.3 ± 0.8 | 0.3 ± 0.3 | 0.4 ± 0.3 | 3.3 ± 1.4 |
| *Betula nana* | 0.0 ± 0.0 | 0.4 ± 0.3 | 0.0 ± 0.0 | 0.3 ± 0.3 | 0.0 ± 0.0 | 0.3 ± 0.3 | 0.0 ± 0.0 |
| unidentified deciduous species | 0.0 ± 0.0 | 0.1 ± 0.1 | 0.0 ± 0.0 | 0.0 ± 0.0 | 0.1 ± 0.1 | 0.0 ± 0.0 | 0.0 ± 0.0 |
| **Graminoids** |  |  |  |  |  |  |  |
| *Deschampsia flexuosa* | 18.2 ± 6.1 | 0.0 ± 0.0 | 2.1 ± 1.5 | 5.7 ± 3.7 | 6.0 ± 3.8 | 4.3 ± 3.9 | 11.1 ± 6.7 |
| *Carex vaginata* | 0.0 ± 0.0 | 0.0 ± 0.0 | 0.7 ± 0.7 | 0.0 ± 0.0 | 0.0 ± 0.0 | 0.0 ± 0.0 | 1.0 ± 1.0 |
| **Forbs** |  |  |  |  |  |  |  |
| *Rubus chamaemorus* | 4.2 ± 2.4 | 2.8 ± 0.9 | 0.0 ± 0.0 | 1.0 ± 0.7 | 5.1 ± 3.1 | 1.1 ± 0.8 | 2.1 ± 0.8 |
| *Conus suecica* | 11.3 ± 2.7 | 0.0 ± 0.0 | 0.0 ± 0.0 | 4.4 ± 2.4 | 1.1 ± 1.0 | 1.3 ± 1.0 | 8.2 ± 3.4 |
| *Pedicularis lapponica* | 0.3 ± 0.2 | 0.0 ± 0.0 | 0.0 ± 0.0 | 0.0 ± 0.0 | 0.1 ± 0.1 | 0.3 ± 0.2 | 0.0 ± 0.0 |
| **Mosses** |  |  |  |  |  |  |  |
| *Pleurozium schreberi* | 68.8 ± 5.4 | 3.8 ± 2.5 | 40.0 ± 6.5 | 39.7 ± 8.8 | 36.8 ± 8.9 | 39.9 ± 9.3 | 33.6 ± 10.0 |
| *Polytrichum commune* | 0.6 ± 0.4 | 2.6 ± 1.2 | 2.6 ± 0.7 | 1.1 ± 0.5 | 2.1 ± 0.8 | 2.6 ± 1.6 | 1.9 ± 0.7 |
| *Dicranum* spp. | 10.6 ± 2.5 | 8.8 ± 3.5 | 23.4 ± 5.3 | 19.3 ± 5.8 | 8.3 ± 2.5 | 16.1 ± 6.0 | 13.3 ± 4.1 |
| *Hylocomium splendens* | 4.2 ± 2.4 | 0.0 ± 0.0 | 0.1 ± 0.1 | 0.8 ± 0.8 | 3.8 ± 3.1 | 1.0 ± 0.7 | 0.1 ± 0.1 |
| Unidentified moss | 0.2 ± 0.1 | 0.6 ± 0.5 | 0.1 ± 0.1 | 0.1 ± 0.1 | 0.0 ± 0.0 | 0.1 ± 0.1 | 1.0 ± 0.7 |
| *Sphagnum* sp*.* | 0.0 ± 0.0 | 0.4 ± 0.4 | 0.0 ± 0.0 | 0.0 ± 0.0 | 0.0 ± 0.0 | 0.0 ± 0.0 | 0.6 ± 0.6 |
| **Lichens** |  |  |  |  |  |  |  |
| *Nephroma arcticum* | 0.0 ± 0.0 | 0.0 ± 0.0 | 15.3 ± 4.0 | 6.1 ± 4.0 | 9.4 ± 4.1 | 2.4 ± 2.0 | 2.5 ± 2.1 |
| *Cladonia* spp. | 0.5 ± 0.3 | 4.4 ± 1.6 | 14.9 ± 3.6 | 5.4 ± 2.4 | 3.8 ± 1.6 | 4.3 ± 2.6 | 12.9 ± 4.5 |
| *Cladonia arbuscular* | 0.0 ± 0.0 | 0.6 ± 0.3 | 8.1 ± 1.2 | 3.5 ± 1.6 | 2.2 ± 1.0 | 2.1 ± 1.0 | 3.9 ± 1.5 |
| *Stereocaulon paschale* | 0.0 ± 0.0 | 0.0 ± 0.0 | 10.7 ± 3.2 | 6.3 ± 3.6 | 0.3 ± 0.2 | 5.3 ± 2.9 | 2.5 ± 1.6 |
| *Thamnolia vermicularis* | 2.1 ± 1.3 | 0.0 ± 0.0 | 1.1 ± 0.7 | 3.6 ± 1.8 | 0.3 ± 0.3 | 0.3 ± 0.3 | 0.1 ± 0.1 |
| Unidentified lichen | 0.0 ± 0.0 | 0.1 ± 0.1 | 0.0 ± 0.0 | 0.0 ± 0.0 | 0.0 ± 0.0 | 0.1 ± 0.1 | 0.0 ± 0.0 |
| **Vascular cryptogram** |  |  |  |  |  |  |  |
| *Selaginella selagonoides* | 0.0 ± 0.0 | 0.0 ± 0.0 | 1.0 ± 1.0 | 0.0 ± 0.0 | 0.0 ± 0.0 | 1.4 ± 1.4 | 0.0 ± 0.0 |
| **Standing dead** | 14.8 ± 2.6 | 29.3 ± 3.4 | 17.9 ± 2.8 | 24.2 ± 4.5 | 19.9 ± 4.0 | 20.7 ± 3.0 | 17.9 ± 3.4 |
| **Litter** | 2.7 ± 1.1 | 20.9 ± 2.4 | 0.6 ± 0.3 | 6.3 ± 2.5 | 7.6 ± 3.0 | 11.0 ± 3.8 | 7.9 ± 2.7 |
| **Bare soil** | 0.3 ± 0.3 | 10.7 ± 2.0 | 0.0 ± 0.0 | 5.0 ± 2.1 | 4.2 ± 2.2 | 1.9 ± 1.0 | 3.6 ± 2.0 |

Table S5. Results of simple main effect (SME) tests for principal component (PC) score data on plant functional groups. Score data (PC2) was obtained from principal component analysis i.e., tested variables represent scores for % coverage of the plant functional groups; deciduous shrubs, lichens, and litter + bare soil (PC2) in 2020 and of graminoids, forbs, and lichens (PC2) in 2021. Abbreviations: W × S = warming × site interaction, -PAR × S = increased cloudiness × site interaction, W × -PAR × S = warming × increased cloudiness × site interactions, T1 = tundra1 site, T2 = tundra2 site, W+ = warming treatments (i.e., warming alone and warming + increased cloudiness), W- = treatments without warming (i.e., control and increased cloudiness alone), -PAR+ = increased cloudiness treatments (i.e., increased cloudiness alone and warming + increased cloudiness), -PAR- = treatments without increased cloudiness/thin cloud cover (i.e., control and warming alone treatments). Only statistically significant (*P*-value ≤ 0.05) are shown (*n* = 20 per site, *n* = 10 for W × S and -PAR × S, and *n* = 15 for W × -PAR × S interactions).

| Year | Plant functional groups | Tested variable | Main/Interaction effects | SME comparison | SME test *P*-value |
| --- | --- | --- | --- | --- | --- |
| 2020 |  |  |  |  |  |
|  | Deciduous shrubs, lichens, and litter + bare soil | PC2 | W × S | W+ vs W- in T2 | 0.009 |
|  |  |  | -PAR × S | -PAR+ vs -PAR- in T2 | 0.001 |
| 2021 |  |  |  |  |  |
|  | graminoids, forbs, and lichens | PC2 | W × -PAR × S | -PAR+ vs -PAR- in W+ and T1 | 0.002 |
|  |  |  |  | W+ vs W- in -PAR+ and T1 | 0.010 |

Table S6. Vegetation coverage (%, mean ± S.E, *n* = 5 per site per treatment) of plant species functional groups; Deciduous shrubs, lichens, and litter and bare soil, and of plant species; *Vaccinium myrtillus* (VM), *Vaccinium uliginosum* (VU), *Betula nana* (BN), *Nephroma arcticum* (NA), *Cladonia* spp. (Cspp.), *Cladonia arbuscular* (CA), *Stereocaulon paschale* (SP), and *Thamnolia vermicularis* (TV) for tundra1 (T1), palsa (P) and tundra2 (T2) sites in control (C), warming (W), increased cloudiness (-PAR), and warming + increased cloudiness (W-PAR) treatments in 2020.

| Treatments × site | | Deciduous shrubs | Lichens | Litter + bare soil | VM | VU | BN | NA | Csp. | CA | SP | TV |
| --- | --- | --- | --- | --- | --- | --- | --- | --- | --- | --- | --- | --- |
| C | T1 | 14.4 ± 4.8 | 1.0 ± 0.6 | 15.2 ± 15.0 | 14.0 ± 4.8 | 0.0 ± 0.0 | 0.4 ± 0.4 | 0.0 ± 0.0 | 0.0 ± 0.0 | 0.0 ± 0.0 | 0.0 ± 0.0 | 1.0 ± 0.6 |
|  | P | 0.8 ± 0.6 | 2.2 ± 2.0 | 28.6 ± 17.0 | 0.6 ± 0.4 | 0.0 ± 0.0 | 0.2 ± 0.2 | 0.0 ± 0.0 | 2.0 ± 2.0 | 0.2 ± 0.2 | 0.0 ± 0.0 | 0.0 ± 0.0 |
|  | T2 | 20.8 ± 4.7 | 26.0 ± 12.1 | 0.0 ± 0.0 | 20.0 ± 4.7 | 0.6 ± 0.4 | 0.2 ± 0.2 | 8.4 ± 5.4 | 2.4 ± 1.1 | 3.2 ± 1.9 | 11.2 ± 9.7 | 0.8 ± 0.4 |
| W | T1 | 10.2 ± 4.0 | 0.4 ± 0.4 | 0.0 ± 0.0 | 10.2 ± 4.1 | 0.0 ± 0.0 | 0.0 ± 0.0 | 0.2 ± 0.2 | 0.2 ± 0.2 | 0.0 ± 0.0 | 0.0 ± 0.0 | 0.0 ± 0.0 |
|  | P | 1.0 ± 1.0 | 1.8 ± 1.2 | 0.4 ± 0.4 | 1.0 ± 1.0 | 0.0 ± 0.0 | 0.0 ± 0.0 | 0.0 ± 0.0 | 1.2 ± 1.0 | 0.4 ± 0.4 | 0.2 ± 0.2 | 0.0 ± 0.0 |
|  | T2 | 8.0 ± 2.0 | 11.8 ± 4.0 | 0.0 ± 0.0 | 8.0 ± 2.0 | 0.0 ± 0.0 | 0.0 ± 0.0 | 9.4 ± 4.1 | 0.4 ± 0.2 | 1.4 ± 0.4 | 0.6 ± 0.2 | 0.0 ± 0.0 |
| -PAR | T1 | 5.0 ± 1.6 | 0.2 ± 0.2 | 0.4 ± 0.2 | 5.0 ± 1.6 | 0.0 ± 0.0 | 0.0 ± 0.0 | 0.2 ± 0.2 | 0.0 ± 0.0 | 0.0 ± 0.0 | 0.0 ± 0.0 | 0.0 ± 0.0 |
|  | P | 0.2 ± 0.2 | 0.6 ± 0.4 | 1.4 ± 1.0 | 0.0 ± 0.0 | 0.0 ± 0.0 | 0.2 ± 0.2 | 0.0 ± 0.0 | 0.6 ± 0.4 | 0.0 ± 0.0 | 0.0 ± 0.0 | 0.0 ± 0.0 |
|  | T2 | 6.0 ± 1.0 | 4.6 ± 1.4 | 0.0 ± 0.0 | 6.0 ± 1.0 | 0.0 ± 0.0 | 0.0 ± 0.0 | 0.8 ± 0.5 | 1.8 ± 0.9 | 0.0 ± 0.0 | 2.0 ± 0.8 | 0.0 ± 0.0 |
| W-PAR | T1 | 5.0 ± 2.5 | 0.4 ± 0.2 | 0.2 ± 0.2 | 5.0 ± 2.5 | 0.0 ± 0.0 | 0.0 ± 0.0 | 0.0 ± 0.0 | 0.2 ± 0.2 | 0.0 ± 0.0 | 0.0 ± 0.0 | 0.2 ± 0.2 |
|  | P | 1.0 ± 1.0 | 0.8 ± 0.4 | 0.8 ± 0.5 | 1.0 ± 1.0 | 0.0 ± 0.0 | 0.0 ± 0.0 | 0.0 ± 0.0 | 0.8 ± 0.4 | 0.0 ± 0.0 | 0.0 ± 0.0 | 0.0 ± 0.0 |
|  | T2 | 4.0 ± 0.9 | 5.2 ± 1.3 | 0.2 ± 0.2 | 3.6 ± 0.7 | 0.4 ± 0.4 | 0.0 ± 0.0 | 0.4 ± 0.4 | 4.0 ± 1.6 | 0.2 ± 0.2 | 0.4 ± 0.4 | 0.2 ± 0.2 |

Table S7. Vegetation coverage (%, mean ± S.E, n = 5 per site per treatment) of plant species functional groups; Graminoids, forbs, lichens, and of plant species; *Deschampsia flexuosa* (DF)*, Carex vaginata* (CV)*, Rubus chamaemorus* (RC)*, Conus suecica* (CS)*, Pedicularis lapponica* (PL)*, Nephroma arcticum* (NA), *Cladonia* spp. (Cspp.), *Cladonia arbuscular* (CA), *Stereocaulon paschale* (SP), and *Thamnolia vermicularis* (TV) for tundra1 (T1), palsa (P) and tundra2 (T2) sites in control (C), warming (W), increased cloudiness (-PAR), and warming + increased cloudiness (W-PAR) treatments in 2021.

| Treatments × site | | Graminoids | forbs | lichens | DF | CV | RC | CS | PL | NA | CR | CA | SP | TV |
| --- | --- | --- | --- | --- | --- | --- | --- | --- | --- | --- | --- | --- | --- | --- |
| C | T1 | 15.8 ± 10.0 | 13.3 ± 5.5 | 7.9 ± 4.9 | 15.8 ± 10.0 | 0.0 ± 0.0 | 0.0 ± 0.0 | 13.3 ± 5.5 | 0.0 ± 0.0 | 0.0 ± 0.0 | 0.4 ± 0.4 | 0.0 ± 0.0 | 0.0 ± 0.0 | 7.5 ± 4.8 |
|  | P | 0.0 ± 0.0 | 2.1 ± 1.3 | 1.7 ± 1.7 | 0.0 ± 0.0 | 0.0 ± 0.0 | 2.9 ± 2.0 | 0.0 ± 0.0 | 0.0 ± 0.0 | 0.0 ± 0.0 | 1.7 ± 1.7 | 0.0 ± 0.0 | 0.0 ± 0.0 | 0.0 ± 0.0 |
|  | T2 | 1.3 ± 1.3 | 0.0 ± 0.0 | 65.0 ± 13.2 | 1.3 ± 1.3 | 0.0 ± 0.0 | 0.0 ± 0.0 | 0.0 ± 0.0 | 0.0 ± 0.0 | 18.3 ± 10.4 | 14.2 ± 5.1 | 10.4 ± 3.0 | 18.8 ± 8.9 | 3.3 ± 2.4 |
| W | T1 | 11.3 ± 10.2 | 14.2 ± 6.0 | 2.5 ± 1.0 | 11.3 ± 10.2 | 0.0 ± 0.0 | 13.3 ± 8.6 | 3.3 ± 2.8 | 0.4 ± 0.4 | 0.0 ± 0.0 | 1.7 ± 1.0 | 0.0 ± 0.0 | 0.0 ± 0.0 | 0.8 ± 0.8 |
|  | P | 0.0 ± 0.0 | 2.1 ± 1.6 | 4.6 ± 3.0 | 0.0 ± 0.0 | 0.0 ± 0.0 | 2.1 ± 1.6 | 0.0 ± 0.0 | 0.0 ± 0.0 | 0.0 ± 0.0 | 3.8 ± 2.8 | 0.8 ± 0.8 | 0.0 ± 0.0 | 0.0 ± 0.0 |
|  | T2 | 6.7 ± 5.7 | 0.0 ± 0.0 | 40.8 ± 5.3 | 6.7 ± 5.7 | 0.0 ± 0.0 | 0.0 ± 0.0 | 0.0 ± 0.0 | 0.0 ± 0.0 | 28.3 ± 6.8 | 5.8 ± 3.9 | 5.8 ± 2.0 | 0.8 ± 0.5 | 0.0 ± 0.0 |
| -PAR | T1 | 12.5 ± 11.5 | 4.6 ± 3.0 | 0.0 ± 0.0 | 12.5 ± 11.5 | 0.0 ± 0.0 | 0.0 ± 0.0 | 3.8 ± 2.7 | 0.8 ± 0.5 | 0.0 ± 0.0 | 0.0 ± 0.0 | 0.0 ± 0.0 | 0.0 ± 0.0 | 0.0 ± 0.0 |
|  | P | 0.0 ± 0.0 | 3.3 ± 2.1 | 3.3 ± 1.6 | 0.0 ± 0.0 | 0.0 ± 0.0 | 3.3 ± 2.1 | 0.0 ± 0.0 | 0.0 ± 0.0 | 0.0 ± 0.0 | 2.1 ± 0.9 | 0.8 ± 0.8 | 0.0 ± 0.0 | 0.0 ± 0.0 |
|  | T2 | 0.4 ± 0.4 | 0.0 ± 0.0 | 40.0 ± 12.6 | 0.4 ± 0.4 | 0.0 ± 0.0 | 0.0 ± 0.0 | 0.0 ± 0.0 | 0.0 ± 0.0 | 7.1 ± 5.7 | 10.8 ± 7.4 | 5.4 ± 2.1 | 15.8 ± 6.8 | 0.8 ± 0.8 |
| W-PAR | T1 | 33.3 ± 16.8 | 27.1 ± 5.6 | 0.0 ± 0.0 | 33.3 ± 16.8 | 0.0 ± 0.0 | 3.3 ± 1.9 | 24.6 ± 4.7 | 0.0 ± 0.0 | 0.0 ± 0.0 | 0.0 ± 0.0 | 0.0 ± 0.0 | 0.0 ± 0.0 | 0.0 ± 0.0 |
|  | P | 0.0 ± 0.0 | 2.9 ± 1.6 | 10.8 ± 5.8 | 0.0 ± 0.0 | 0.0 ± 0.0 | 2.9 ± 1.6 | 0.0 ± 0.0 | 0.0 ± 0.0 | 0.0 ± 0.0 | 10.0 ± 5.4 | 0.8 ± 0.5 | 0.0 ± 0.0 | 0.0 ± 0.0 |
|  | T2 | 2.9 ± 2.9 | 0.0 ± 0.0 | 55.0 ± 6.6 | 0.0 ± 0.0 | 2.9 ± 2.9 | 0.0 ± 0.0 | 0.0 ± 0.0 | 0.0 ± 0.0 | 7.5 ± 6.0 | 28.8 ± 8.7 | 10.8 ± 2.0 | 7.5 ± 4.0 | 0.4 ± 0.4 |

Table S8. Simple main effect (SME) test output results for leaf anatomy, leaf carbon (C), and chlorophyll content variables of *E. hermaphroditum*, *V. myrtillus* and *V. vitis-idaea*. Abbreviations: W = warming, -PAR = increased cloudiness, W × -PAR = warming × increased cloudiness interaction. W+ = warming treatments (i.e., warming alone and warming + increased cloudiness), W- = treatments without warming (i.e., control and increased cloudiness alone), -PAR+ = increased cloudiness treatments (i.e., increased cloudiness alone and warming + increased cloudiness), -PAR- = treatments without increased cloudiness (i.e., control and warming alone treatments), ↓ indicates a decreasing effect and ↑ indicates an increasing effect of the tested factor. Only statistically significant (*P*-value ≤ 0.05) are shown (*n* = 12).

| Plant Species | Tested variable | Interaction | SME comparison | SME test *P*-value | Change |
| --- | --- | --- | --- | --- | --- |
| *V. myrtillus* |  |  |  |  |  |
|  | Upper epidermis thickness (µm) | W × -PAR | W+ vs W- in -PAR+ | 0.003 | ↓ -11.5 % |
|  | Lower epidermis thickness (µm) | W × -PAR | W+ vs W- in -PAR+ | <0.001 | ↓ -12.5 % |
|  |  | W × -PAR | -PAR+ vs -PAR- in W- | <0.001 | ↑ 13.0 % |
|  | Palisade parenchyma thickness (µm) | W × -PAR | -PAR+ vs -PAR- in W+ | 0.009 | ↓ -22.5 % |
|  | Leaf C content | W × -PAR | -PAR+ vs -PAR- in W- | 0.015 | ↑ 3.0 % |
|  |  | W × -PAR | W+ vs W- in -PAR+ | 0.029 | ↓ -2.6 % |
| *V. vitis-idaea* |  |  |  |  |  |
|  | Leaf thickness (µm) | W × -PAR | W+ vs W- in -PAR- | 0.021 | ↓ -20.6 % |
| *E. hermaphroditum* |  |  |  |  |  |
|  | Chlorophyll content (mgm^-2^) | W × -PAR | W+ vs W- in -PAR+ | <0.001 | ↑ 38.5 % |
|  |  | W × -PAR | -PAR+ vs -PAR- in W+ | 0.050 | ↑ 16.2 % |

PC1 (35.5 %)

Fig. S1. (a) The principal component (PC1 and PC2) scores (means ± S.Es) and (b) their corresponding loading variables for percentage coverage of plant species functional groups, and litter and bare soil. Analysis was done on mesocosms after their collection in late growing season 2019 and before their exposure to experimental treatments the following year 2020. Therefore, significant site differences here indicate variations that appeared naturally between the different studied sites. The variation explained by each PC is shown in parentheses. Abbreviations: S = site, T1 = tundra1 site, T2 = tundra2 site, P = palsa site. The univariate ANOVA *P*-value for statistically significant (*P* ≤ 0.05) site differences are shown in the figures (*n* = 20 per sites).

Figure S2: Hourly mean air temperature and photosynthetic active radiation (PAR) based on temperature (2010-2019) averages and cloud simulations from North Sweden (68°24′45″N, 18°08′13″E) were used during June week 1 in (a) control (C), (b) warming (W), (c) increased cloudiness (-PAR), and (d) warming + increased cloudiness (W-PAR) treatments.

Figure S3: Hourly mean air temperature and photosynthetic active radiation (PAR) based on temperature (2010-2019) averages and cloud simulations from North Sweden (68°24′45″N, 18°08′13″E) were used during June week 2 in (a) control (C), (b) warming (W), (c) increased cloudiness (-PAR), and (d) warming + increased cloudiness (W-PAR) treatments.

Figure S4: Hourly mean air temperature and photosynthetic active radiation (PAR) based on temperature (2010-2019) averages and cloud simulations from North Sweden (68°24′45″N, 18°08′13″E) were used during June week 3 in (a) control (C), (b) warming (W), (c) increased cloudiness (-PAR), and (d) warming + increased cloudiness (W-PAR) treatments.

Figure S5: Hourly mean air temperature and photosynthetic active radiation (PAR) based on temperature (2010-2019) averages and cloud simulations from North Sweden (68°24′45″N, 18°08′13″E) were used during June week 4 in (a) control (C), (b) warming (W), (c) increased cloudiness (-PAR), and (d) warming + increased cloudiness (W-PAR) treatments.

Figure S6: Hourly mean air temperature and photosynthetic active radiation (PAR) based on temperature (2010-2019) averages and cloud simulations from North Sweden (68°24′45″N, 18°08′13″E) were used during July week 1 in (a) control (C), (b) warming (W), (c) increased cloudiness (-PAR), and (d) warming + increased cloudiness (W-PAR) treatments.

Figure S7: Hourly mean air temperature and photosynthetic active radiation (PAR) based on temperature (2010-2019) averages and cloud simulations from North Sweden (68°24′45″N, 18°08′13″E) were used during July week 2 in (a) control (C), (b) warming (W), (c) increased cloudiness (-PAR), and (d) warming + increased cloudiness (W-PAR) treatments.

Figure S8: Hourly mean air temperature and photosynthetic active radiation (PAR) based on temperature (2010-2019) averages and cloud simulations from North Sweden (68°24′45″N, 18°08′13″E) were used during July week 3 in (a) control (C), (b) warming (W), (c) increased cloudiness (-PAR), and (d) warming + increased cloudiness (W-PAR) treatments.

Figure S9: Hourly mean air temperature and photosynthetic active radiation (PAR) based on temperature (2010-2019) averages and cloud simulations from North Sweden (68°24′45″N, 18°08′13″E) were used during July week 4 in (a) control (C), (b) warming (W), (c) increased cloudiness (-PAR), and (d) warming + increased cloudiness (W-PAR) treatments.

Figure S10: Hourly mean air temperature and photosynthetic active radiation (PAR) based on temperature (2010-2019) averages and cloud simulations from North Sweden (68°24′45″N, 18°08′13″E) were used during August week 1 in (a) control (C), (b) warming (W), (c) increased cloudiness (-PAR), and (d) warming + increased cloudiness (W-PAR) treatments.

Figure S11: Hourly mean air temperature and photosynthetic active radiation (PAR) based on temperature (2010-2019) averages and cloud simulations from North Sweden (68°24′45″N, 18°08′13″E) were used during August week 2 in (a) control (C), (b) warming (W), (c) increased cloudiness (-PAR), and (d) warming + increased cloudiness (W-PAR) treatments.

Figure S12: Hourly mean air temperature and photosynthetic active radiation (PAR) based on temperature (2010-2019) averages and cloud simulations from North Sweden (68°24′45″N, 18°08′13″E) were used during August week 3 in (a) control (C), (b) warming (W), (c) increased cloudiness (-PAR), and (d) warming + increased cloudiness (W-PAR) treatments.

Figure S13: Hourly mean air temperature and photosynthetic active radiation (PAR) based on temperature (2010-2019) averages and cloud simulations from North Sweden (68°24′45″N, 18°08′13″E) were used during August week 4 in (a) control (C), (b) warming (W), (c) increased cloudiness (-PAR), and (d) warming + increased cloudiness (W-PAR) treatments.

PC1 (27.6 %)

PC2 (21.3 %)

Fig. S14. (a&b). The principal component (PC) scores (means ± S.Es) and (c) their corresponding loading variables for percentage coverage of plant species functional groups, and litter and bare soil in 2020. The variation explained by each PC is shown in parentheses. Abbreviations: C = control (ambient warming and PAR/thin cloud), W = warming, -PAR = increased cloudiness (thick cloud), W-PAR = warming + increased cloudiness, T1C = T1 site in control, T1W = T1 site in warming, T1-PAR = T1 site in increased cloudiness, T1W-PAR = T1 site in warming + increased cloudiness, T2C = T2 site in control, T2W = T2 site in warming, T2-PAR = T2 site in increased cloudiness, T2W-PAR = T2 site in warming + increased cloudiness, PC = palsa site in control, PW = palsa site in warming, P-PAR = palsa site in increased cloudiness, PW-PAR = palsa site in warming + increased cloudiness treatments, W × S = warming × site, -PAR × S = increased cloudiness × site. The univariate ANOVA *P*-values for statistically significant (*P* ≤ 0.05) and for marginally statistically significant (*P* ≤ 0.1) main and interaction effects are shown in the figures (*n* = 5 per site per treatment, *n* = 15 per treatments).

Fig S15. Total biomass (g, mean ± SE, *n* = 6-11 per treatment) of (a) *V. myrtillus*, (b) *V. vitis-idaea*, and (c) *R. chamaemorus* from mesocosms in control (C), warming (W), increased cloudiness (-PAR), and warming + increased cloudiness (W-PAR) treatments. *P*-values from univariate ANOVA are shown. **P* values from Kruskal-wallis test. Note the different y-axes scales.

Fig S16. Total biomass (g, mean ± SE, *n* = 15 per treatment) of (a) other evergreen shrubs, (b) other deciduous species, (c) lichens, (d) mosses, (e) graminoids, and (f) litter from mesocosms in control (C), warming (W), increased cloudiness (-PAR), and warming + increased cloudiness (W-PAR) treatments. *P* values from Kruskal-wallis non-parametric tests are shown. Note the different y-axes scales.

Fig S17. Total biomass (g, mean ± SE, *n* = 15 per treatment) of (a) Standing dead, and (b) roots and other non-photosynthetic plant parts from mesocosms in control (C), warming (W), increased cloudiness (-PAR), and warming + increased cloudiness (W-PAR) treatments. *P* values from Kruskal-wallis non-parametric tests are shown. Note the different y-axes scales. Standing dead = dead plants with root connections.

Fig S18. Greenness index (nG) variability during 8 measurement campaigns in (a) control (C), warming (W), increased cloudiness (-PAR), and warming + increased cloudiness (W-PAR) treatments averaged across all sites (mean, *n* = 15 per treatment), and (b) tundra1 (T1), tundra2 (T2) and palsa (P) sites under increased cloudiness treatments (mean, *n =* 5 per site, *n* = 30 per treatment). (C) Mean nG (±SE) across the growing season averaged across all sites per treatment. W × T = warming × time, -PAR × T = increased cloudiness × time, and -PAR × S × T = increased cloudiness × site × time interactions. T1C = T1 site in control, T1-PAR = T1 site in increased cloudiness, T2C = T2 site in control, T2-PAR = T2 site in increased cloudiness, PC = P site in control, P-PAR = P site in increased cloudiness treatments. Note the different y-axis scale in (c).

Fig S19. Leaf chlorophyll content (mgm^-2^, mean ± SE, *n* = 6-11 per treatment) of (a) *V. myrtillus*, (b) *V. vitis-idaea*, and (c) *R. chamaemorus* from mesocosms in control (C), warming (W), increased cloudiness (-PAR), and warming + increased cloudiness (W-PAR) treatments. *P* values from univariate ANOVA are shown. Note the different y-axes scales.

Fig S20. (a) Leaf C and (b) Leaf N content (%, mean ± SE, *n* = 6 per treatment) of *E. hermaphroditum* and *V. myrtillus*, respectively from mesocosms in control (C), warming (W), increased cloudiness (-PAR), and warming + increased cloudiness (W-PAR) treatments. *P* values from univariate ANOVA are shown. **P* values from Kruskal-wallis test.

References

Aphalo, P., J. (2015). The r4photobiology suite. UV4Plants Bulletin 1:21-9.

Both, A., J., Bugbee, B., Kubota et al. (2017). Proposed product label for electric lamps used in the plant sciences. *Horttechnology*, 27(4):544-9. https://doi.org/10.21273/HORTTECH03648-16.

ISO. Space environment (natural and artificial) - Process for determining solar irradiances. ISO Standard 21348:2007.

R Core Team. (2017). R: A Language and Environment for Statistical Computing. R Foundation for Statistical Computing, Vienna, Austria.
